# *SLC10A7* mutations cause a skeletal dysplasia with amelogenesis imperfecta mediated by GAG biosynthesis defects

**DOI:** 10.1038/s41467-018-05191-8

**Published:** 2018-08-06

**Authors:** Johanne Dubail, Céline Huber, Sandrine Chantepie, Stephan Sonntag, Beyhan Tüysüz, Ercan Mihci, Christopher T. Gordon, Elisabeth Steichen-Gersdorf, Jeanne Amiel, Banu Nur, Irene Stolte-Dijkstra, Albertien M. van Eerde, Koen L. van Gassen, Corstiaan C. Breugem, Alexander Stegmann, Caroline Lekszas, Reza Maroofian, Ehsan Ghayoor Karimiani, Arnaud Bruneel, Nathalie Seta, Arnold Munnich, Dulce Papy-Garcia, Muriel De La Dure-Molla, Valérie Cormier-Daire

**Affiliations:** 1 Department of Genetics, INSERM UMR 1163, Université Paris Descartes-Sorbonne Paris Cité, Institut Imagine, AP-HP, Hôpital Necker Enfants Malades, 75015 Paris, France; 2 Cell Growth and Tissue Repair CRRET Laboratory, Université Paris-Est Créteil, EA 4397 CNRS 9215, Créteil, F-94010 France; 3 PolyGene AG, Rümlang, CH-8153, Switzerland; 4 Department of Pediatric Genetics, Cerrahpasa Medicine School, Istanbul University, 34290 Istanbul, Turkey; 5 Akdeniz University Paediatric Genetic Deaprtment, 07059 Antalya, Turkey; 6 Laboratory of Embryology and Genetics of Congenital Malformations, INSERM UMR 1163, Institut Imagine, 75015 Paris, France; 7 Department of Paediatrics I, Medical University of Innsbruck, A-6020 Innsbruck, Austria; 8 Department of Genetics, University Medical Center Groningen, University of Groningen, 9700 Groningen, The Netherlands; 9 Department of Genetics, Center for Molecular Medicine, University Medical Center Utrecht, 3508 Utrecht, The Netherlands; 10 Division of Paediatric Plastic Surgery, Wilhelmina Children´s Hopsital, 3584 Utrecht, The Netherlands; 11 Department of Human Genetics, Radboud University Medical Center, 6525 Nijmegen, The Netherlands; 12 Department of Clinical Genetics, Maastricht University Medical Center, 6202 Maastricht, The Netherlands; 13 Institute of Human Genetics, Julius Maximilians University Würzburg, 97074 Würzburg, Germany; 14 Genetics Research Centre, Molecular and Clinical Sciences Institute, St George’s, University of London, Cranmer Terrace, London SW17 ORE, UK; 15 Next Generation Genetic Clinic, 9175954353 Mashhad, Iran; 16 Razavi Cancer Research Center, Razavi Hospital, Imam Reza International University, 9198613636 Mashhad, Iran; 17 AP-HP, Biochimie Métabolique et cellulaire, Hôpital Bichat, 75018 Paris, France; 18 Laboratory of Molecular Oral Pathophysiology, Centre de Recherche des Cordeliers, INSERM UMRS 1138, University Paris-Descartes, University Pierre et Marie Curie-Paris, 75006 Paris, France

## Abstract

Skeletal dysplasia with multiple dislocations are severe disorders characterized by dislocations of large joints and short stature. The majority of them have been linked to pathogenic variants in genes encoding glycosyltransferases, sulfotransferases or epimerases required for glycosaminoglycan synthesis. Using exome sequencing, we identify homozygous mutations in *SLC10A7* in six individuals with skeletal dysplasia with multiple dislocations and amelogenesis imperfecta. *SLC10A7* encodes a 10-transmembrane-domain transporter located at the plasma membrane. Functional studies in vitro demonstrate that *SLC10A7* mutations reduce SLC10A7 protein expression. We generate a *Slc10a7*^*−/−*^ mouse model, which displays shortened long bones, growth plate disorganization and tooth enamel anomalies, recapitulating the human phenotype. Furthermore, we identify decreased heparan sulfate levels in *Slc10a7*^*−/−*^ mouse cartilage and patient fibroblasts. Finally, we find an abnormal *N*-glycoprotein electrophoretic profile in patient blood samples. Together, our findings support the involvement of SLC10A7 in glycosaminoglycan synthesis and specifically in skeletal development.

## Abstract (web summary)

The majority of skeletal dysplasia are caused by pathogenic variants in genes required for glycosaminoglycan (GAG) metabolism. Here, Dubail et al. identify genetic variants in the solute carrier family protein SLC10A7 in families with skeletal dysplasia and amelogenesis imperfecta that disrupt GAG synthesis.

## Introduction

Skeletal dysplasias with multiple dislocations are a group of severe disorders characterized by dislocations of large joints, scoliosis, short stature and a variable combination of cleft palate, heart defects, intellectual disability and obesity^1,2^. More than 10 recessive disorders, including Desbuquois dysplasia and Larsen of Reunion Island syndrome have been described so far^3–5^. With the help of massively parallel sequencing technologies, the majority of these rare disorders have been linked to pathogenic variants in genes encoding glycosyltransferases (“linkeropathies”)^6,7^, sulfotransferases^8,9^, epimerases^10^ or transporters^11^, required for glycosaminoglycan (GAG) biosynthesis^12^. These findings support the existence of a group of inborn errors of development defined by impaired GAG biosynthesis. Pathogenic variants in genes encoding proteins with no known functions have also been implicated in impaired proteoglycan synthesis; e.g., fibroblasts from patients with pathogenic variants of calcium-activated nucleotidase-1^4^ display defective proteoglycan synthesis^13^. These findings suggest that GAG synthesis is more complex than previously described and suggests that there are a number of partners of unknown function still to be identified.

In this study, exome sequencing is performed on a number of patients presenting with skeletal dysplasia with multiple dislocations, identifying six cases of homozygous mutations in *SLC10A7*. This gene encodes a member of the Solute Carrier Family SLC10, which comprises two bile acid carriers, one steroid sulfate transporter and four orphan carriers. SLC10A7 is a 10-transmembrane-domain transporter located at the plasma membrane, with a yet unidentified substrate^14^. Using a deficient mouse model and patient fibroblasts, we demonstrate that SLC10A7 deficiency disrupts GAG biosynthesis and intracellular calcium homoeostasis.

Together, our findings support the involvement of SLC10A7 in GAG biosynthesis and specifically in skeletal and tooth development.

## Results

### Phenotypes of patients with *SLC10A7* mutations

**Fig. 1:**
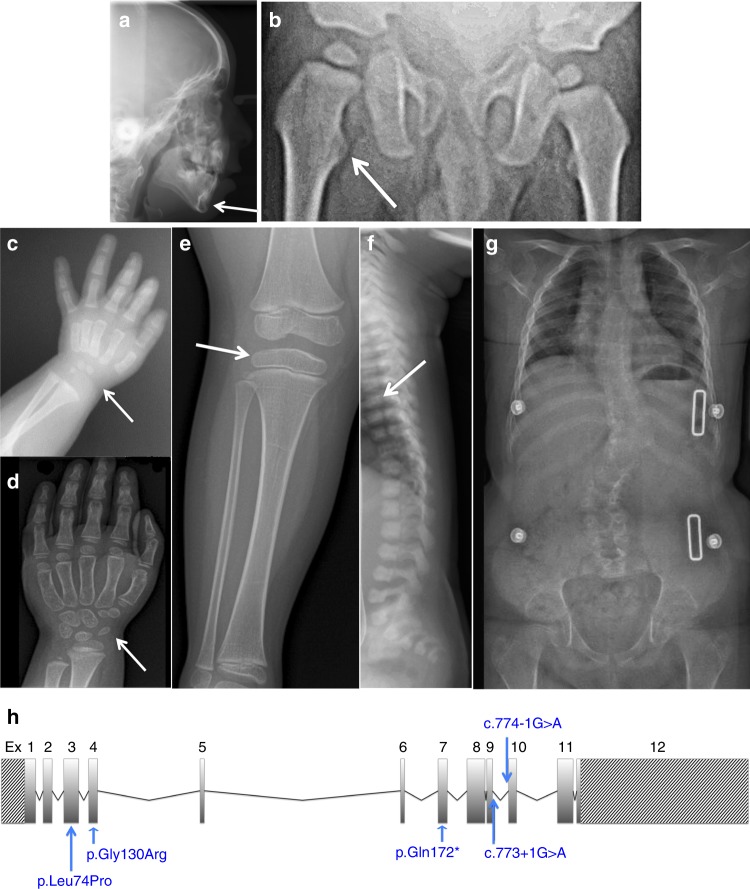
Morphological and skeletal features of *SLC10A7*-deficient patients. **a** Skull X-ray at 6 years of age (Patient 5). Note the retrognathia (arrow). **b** Hip at one year of age (Patient 1). Note the Swedish key appearance of the proximal femur (medial beak and exaggerated trochanters, arrow). **c** Hand X-rays at 6 months of age (Patient 2). **d** Hand X-rays at 3 years of age (Patient 3). Note in (**c**) and (**d**, the advanced carpal ossification (presence of three ossified carpal centres at 6 months and seven ossified carpal centres at 3 years instead of one and three, respectively, see arrows). **e** Knee at 3 years 9 months of age (Patient 3). Note the genu valgum (angled in knee) and flat epiphyses (arrow). **f** Spine X-rays at 1 month of age (Patient 1). Note the coronal clefts and irregular shape of vertebrae (arrow). **g** Spine and hip X-rays at 9 years of age (Patient 4). Note the kyphoscoliosis. Informed consent for publication of images was obtained from all individuals or the legal guardians of minors. **h** Localization of the five SLC10A7 mutations relative to the SLC10A7 gene organization (striped rectangles indicate the 5′ and 3′-UTRs)

The six patients were from six distinct families, originating from Turkey (three patients, consanguineous parents), from the Netherlands (two patients) and from Iran (one patient, consanguineous parents). They presented with pre- and postnatal short stature (< −3 SD), dislocation of large joints, advanced carpal ossification, monkey wrench appearance of the proximal femora in the first months of life, abnormal vertebrae, luxation of knees with genua valga, hyperlordosis or kyphoscoliosis and small epiphyses (Table 1 and Fig. 1). In addition, hypomineralized amelogenesis imperfecta, characterized by yellow–brown enamel with a rough surface, and short and widely spaced tooth crowns, was diagnosed in all six patients (Table 1). Facial abnormalities were present in all patients: a Pierre–Robin sequence (micrognathia, cleft palate and glossoptosis) in two patients, a micrognathia in three other cases and a flat face in the last patient. Additional features observed included a heart defect (one patient), mixed (one patient) and sensorineural hearing loss (one patient), and obesity in the eldest patients. The parents of the Iranian patient have a history of multiple pregnancies that resulted in a spontaneous abortion and neonatal death due to respiratory distress accompanied by micromelia. Two other pregnancies were terminated preterm after the detection of short limbs during ultrasound screening. One of these induced abortions presented an additional cleft palate.

**Table 1:** Clinical features of the six cases

	Family 1	Family 2	Family 3
A. Ethnic origin B. Consanguinity	A. Turkish B. First cousins	A. Turkish B. First cousins	A. Turkish B. Related parents
Parameters and clinical exam at birth	• Term • Length: 44 cm • Weight: 3600 g	• Term • Length: 48 cm • Weight: 1945 g • Head circumference: 33 cm • Hypermobile large joints • Respiratory distress	• Term • Length: 44 cm • Weight: 3106 g • Pierre–Robin sequence • Transient respiratory distress
Clinical features at first exam	At 3 years of age: • Short stature • Short extremities • Round face • Microretrognathia • Hypermobile joints	At 6 months of age: • Length < −3 SD • Round face • Depressed nasal bridge • High palate • Micrognathia • Hypermobile joints	At 3 years of age: • Parameters < P3 • Hypermobile joints • Microretrognathia
Radiological features	• Advanced bone age • Proximal femur “Swedish key” • Short metacarpals and phalanges • Irregular vertebral bodies • Wide metaphyses • Coxa valga	• Advanced carpal ossification • Proximal femur “Swedish key” • Irregular vertebral bodies	• Advanced carpal bone age • Proximal femur “Swedish key” • Small epiphyses
Follow-up: growth and skeleton	5 years: • Height < −3 SD • Knee dislocation • Hyperlordosis • Obesity	2 years: • Height < −3 SD • Knee dislocation • Small epiphyses	4 years: • Height < −5.8 SD • Hyperlordosis • Truncal obesity
Other	• Amelogenesis imperfecta • Tooth agenesis (22 teeth at 13 years of age)	• Amelogenesis imperfecta • Atrial septal defect	• Amelogenesis imperfecta • Speech delay ( >3 years)
	**Family 4**	**Family 5**	**Family 6**
A. Ethnic origin B. Consanguinity	A. Dutch	A. Dutch	A. Iranian B. First cousins
Parameters and clinical exam at birth	• Term • Length: 40.3 cm • Weight: 3530 g • Head circumference: 36 cm • Flat face, short thorax, short extremities	• 39 + 3 • Length: 42.5 cm • Weight: 2680 g • Pierre–Robin sequence respiratory insufficiency (8 days intubation)	• Term • Short extremities • Respiratory distress (ventilator for 1 day)
Clinical features at first exam	At 6 months of age: • Disproportionate short stature, height < −5 SD • Extreme hypermobile knees in hyperextension	8 months: • Disproportionate short stature −3 SD • Genu valgum	
Radiological features	• Advanced carpal and tarsal ossification • Butterfly vertebrae Th11, Th12, horizontal acetabular roof	• Advanced carpal ossification • Proximal femur “Swedish key” • Irregular vertebral bodies and coronal clefts	• Advanced carpal ossification • Short metacarpals (especially of the 4th and 5th finger)
Follow-up: growth and skeleton	10 years: • Height −5 SD • Knee dislocation in extreme valgus • Severe kyphoscoliosis and hyperlordosis • Contractures of the hip	• Sitting height −2.3 SD • OFC 1.87 SD 9 years: • Height: −4.8 SD • Genu valgum • Small epiphyses • Kyphoscoliosis • Hypermobility	At 11 years of age: • Height < −4 SD • Genu valgum • Short extremities • Micrognathia • Hyperlordosis • Patellar instability on the right • Truncal obesity
Other	• Amelogenesis imperfecta	• Amelogenesis imperfecta • Tooth agenesis (Missing teeth: 14 15 24 25 35 44 45) • Mixed hearing loss	• Amelogenesis imperfecta • Tooth agenesis (7 teeth at 12 years of age) • Sensorineural hearing impairment • Decreased visual acuity

### *SLC10A7* mutations and functional analysis

A total of five distinct homozygous *SLC10A7* (GenBank: NM_001317816) mutations were identified (Fig. 1h and Supplementary Fig. 1a). Two were splice site mutations, located in the acceptor site of exon 10 (c.774-1 G > A) and in the donor site of exon 9 (c.773 + 1 G > A), two mutations were a missense substitution located in exon 3 (c.221 T > C [p.Leu74Pro]) and exon 4 (c.388 G > A [p.Gly130Arg]), which were predicted to be damaging by the PolyPhen and Sift algorithms, and one inserted a premature stop codon into exon 7 (c.514 C > T [p.Gln172*]). The two Dutch patients that carried this mutation were genealogically proven to be distantly related through their fathers, while it was not possible to prove a genealogical link between their mothers (Supplementary Fig. 1b). All mutations segregated with the disease (Supplementary Fig. 1b) and were not identified in 200 control chromosomes or public databases.

**Fig. 2:**
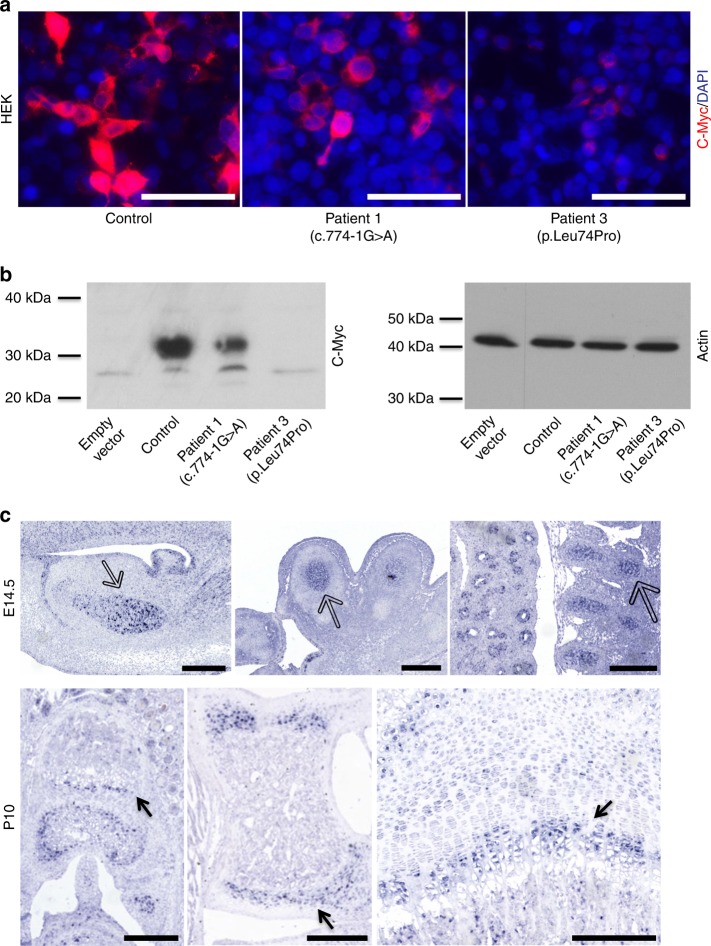
*SLC10A7* mutation consequences and *Slc10a7* tissular expression. **a**, **b** Characterization of wild-type and mutant SLC10A7 proteins. HEK293F cells were transfected with plasmids encoding c-myc tagged wild-type SLC10A7 proteins or c-myc-tagged mutant SLC10A7 proteins from two different patients (Patient 1 and Patient 3). **a** Cells were immunostained with anti-c-Myc antibody (red) and nuclei were counterstained with DAPI (blue). Scale bars = 20 μm. The images are representative of three independent experiments. **b** Total cell lysates were analysed by western blotting using c-Myc antibody. Anti-actin was used as a loading control. The western blot images are cropped from gels that were provided for review as Supplementary Fig. 1d. **c** In situ hybridization analysis of *Slc10a7* mRNA expression in E14.5 mouse embryos and P10 mouse tissues. The blue staining indicates sites of RNA hybridization. At E14.5, empty arrows indicate specific staining in cartilaginous tissues: Meckel cartilage (left panel) in the mandible and phalanges in the digits (central panel) and vertebrae (right panel). Note the positive staining in the lung on the right panel. At P10, filled arrows indicate specific staining in the hypertrophic zone of the growth plate in the digits (left panel), in the tarsals (central panel) and in the epiphysis of the humerus (right panel). Scale bars = 250 μm

*SLC10A7* encodes a 10-transmembrane-domain transporter located at the plasma membrane. Three of the mutations are predicted to be null alleles (affecting essential splice sites or generating a premature stop), whereas the missense variant p.Leu74Pro affects a highly conserved amino acid in the third predicted transmembrane helix. The second missense variant, p.Gly130Arg, also affects a highly conserved amino acid, albeit not residing within a known protein domain. Analyses of complementary DNA from patients carrying *SLC10A7* mutations affecting splice sites identified exon 9 skipping for the c.773 + 1 G > A mutant and an exon 10, and exon 9 and 10 skipping for the c.774-1 G > A mutant. Exon 9 and 10 skipping led to an in-frame deletion of 42 amino acids (Supplementary Fig. 2). We tested the functional consequences of *SLC10A7* mutations using c-myc-tagged wild-type and mutant SLC10A7 (c.774-1 G > A and p.Leu74Pro) in parallel transfections of HEK293F cells. C-myc tagged protein expression was analysed 48 h after transfection. C-myc immunolabelling demonstrated a uniform punctate staining following transfection with wild-type SLC10A7, consistent with a cell membrane localization. A similar expression pattern was observed for all SLC10A7 mutants; however, the level of staining was reduced with the weakest staining being observed with the p.Leu74Pro mutant (Fig. 2a). Similar results were obtained following transfection of COS-1 and MG63 cells (Supplementary Fig. 1b). To confirm the altered expression pattern detected with the SLC10A7 mutants, total HEK293F cell lysates were analysed by western blotting. A significant reduction in the expression of the SLC10A7 mutants compared with wild-type was observed (Fig. 2b). Together, these results demonstrate the deleterious impact of the identified mutations on SLC10A7 protein expression.

### SLC10A7 is expressed in developing skeletal tissues

To correlate the spatio-temporal expression pattern of *SLC10A7* with patient manifestations, in situ hybridization experiments were performed on embryonic and juvenile mouse tissues, i.e., from gestational age 12.5 days (E12.5) to postnatal day 10 (P10). *Slc10a7* mRNA was detected at all the developmental stages studied (Supplementary Fig. 3b), generally localizing to cartilage giving rise to long bones in embryos and in the growth plates of long bones in juvenile mice (Fig. 2c). E12.5 embryos showed the weakest expression, mainly in the heart trabeculae of the developing heart and the cartilage of the vertebrae (Supplementary Fig. 3b). From E14.5 onwards, *Slc10a7* mRNA expression became more ubiquitous, with the strongest expression observed at E16.5 and P0. At E14.5, *Slc10a7* mRNA was strongly expressed in cartilaginous structures such as in the mandible (i.e., Meckel cartilage), in the digits, in the spine and in the lung (Fig. 2c). At P10, *Slc10a7* mRNA expression was localized to the growth plate of several long bones, such as the forefoot digits, the hindfoot tarsals and the humerus, and was more intense in the chondrocytes of the hypertrophic zone. Interestingly, at P0 there was stronger expression in the papillary layer of the oral mucous membrane underneath the palate, as well as in the ameloblast layer of emerging teeth.

To investigate whether the expression patterns observed in mice are also seen in humans, the same experiments were performed on human embryos at 8 and 9 weeks of gestation (Carnegie stages 16 and 19). Similar expression patterns were observed: *SLC10A7* mRNA was detected in the heart and the vertebrae at 8 weeks and in the long-bone cartilage at 9 weeks (Supplementary Fig. 3c).

### *Slc10a7* deficiency causes skeletal dysplasia in mice

**Fig. 3:**
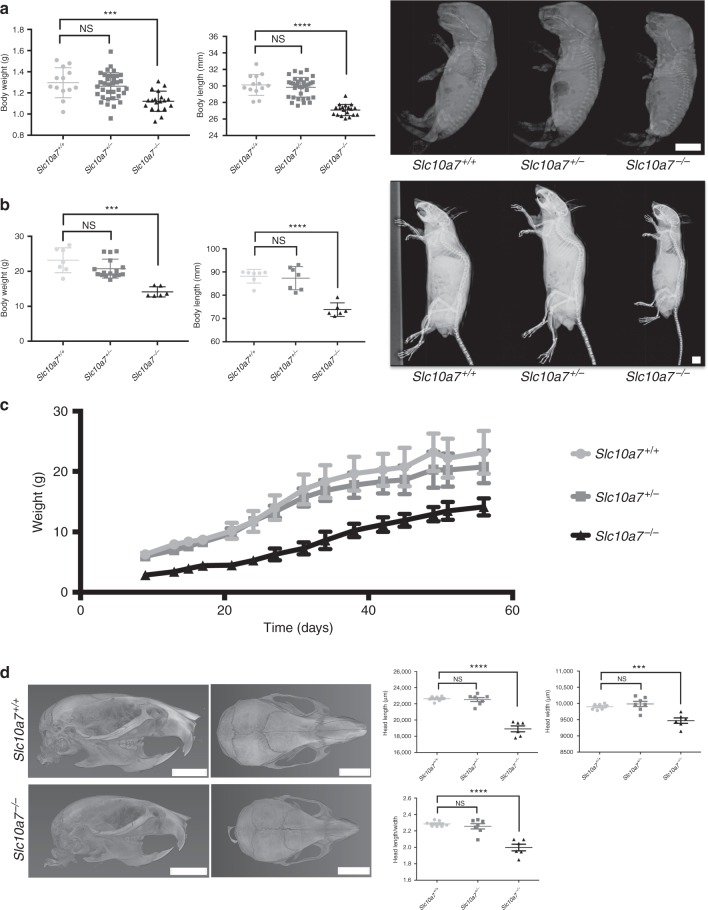
*Slc10a7*^*−/−*^ mice display skeletal dysplasia with skull anomalies. **a**, **b** Measurement of body weight and naso-occipital length (body length) and radiographic assessment of *Slc10a7*^*+/+*^, *Slc10a7*^*+/−*^ and *Slc10a7*^*−/−*^ mice at birth (**a**) or at 8 weeks (**b**), demonstrating that *Slc10a7*^*−/−*^ mice exhibited a skeletal dysplasia with a more rounded skull. **c** Evolution of body weight demonstrating the growth delay in *Slc10a7*^*−/−*^ mice compared with wild-type littermates. **d** Three-dimensional reconstruction of 8-week-old mouse skulls by μCT analysis and skull length and width measurements, demonstrating that *Slc10a7*^*−/−*^ skulls are less elongated than wild-type skulls. Scale bars = 5 mm. Data are expressed as mean ± SD. NS, nonsignificant; ***p ≤ 0.001; ****p ≤ 0.0001 (two-tailed *t-*test). *n* = 13 (*Slc10a7*^*+/+*^), *n* = 35 (*Slc10a7*^*+/−*^) and *n* = 19 (*Slc10a7*^*−/−*^) at birth; *n* = 7 (*Slc10a7*^*+/+*^), *n* = 7 (*Slc10a7*^*+/−*^) and *n* = 6 (*Slc10a7*^*−/−*^) at 8 weeks

In order to decipher the impact of *Slc10a7* deficiency on bone development in vivo, a *Slc10a7*-deficient mouse strain was generated using embryonic stem (ES) cell lines from European Mouse Mutant Cell Repository (EuMMCR) in which a lacZ/*neo* cassette was inserted into intron 1 of *Slc10a7* gene: the resulting heterozygous mice (*Slc10a7*^*+/−*^) were intercrossed to obtain homozygous mice (*Slc10a7*^*−/−*^). At birth, *Slc10a7*^*−/−*^ mice were smaller, displaying significantly reduced body weight (1.121 g ± 0.02188 [*Slc10a7*^*−/−*^] vs. (1.297 g ± 0.03913 [*Slc10a7*^*+/+*^]), reduced naso-occipital length and a more rounded head (as observed with X-ray imaging) compared with wild-type littermates (Fig. 3a). Morphometric measurements indicated that the stylopod (femur and humerus) and zeugopod (tibia and radius) were more affected than the autopod (hindfoot and forefoot) (Supplementary Fig. 4a). Although *Slc10a7*^*−/−*^ hindfeet were smaller, the proportion of ossified tissue, visualized using alizarin red staining, was greater in the tarsals, metatarsals and phalanges of *Slc10a7*^*−/−*^ mice compared with wild-type littermates, suggesting advanced ossification (Supplementary Fig. 4c). The growth delay of *Slc10a7*^*−/−*^ mice continued after birth and at 8 weeks they were smaller than their wild-type littermates (Fig. 3b, c). However, the differences in size reduction between the stylopod/zeugopod and autopod of the *Slc10a7*^*−/−*^ mice was less pronounced than at birth (Supplementary Fig. 4b). No obvious large joint dislocations were visible on the x-rays of *Slc10a7*^*−/−*^ mice at birth or at 8 weeks of age. Skull morphology alterations at 8 weeks was analysed by micro-computed tomography (μCT) analyses. The length and width and the overall elongation (determined from the length/width ratio) of the *Slc10a7*^*−/−*^ skulls was reduced compared with wild-type littermates (Fig. 3d). More precise measurements demonstrated that mostly nasal bone, frontal bone and occipital bone lengths were reduced, while parietal bone length was less reduced and interparietal bone length was not affected in *Slc10a7*^*−/−*^ skulls. Furthermore, only parietal bone and occipital bone widths were slightly smaller in *Slc10a7*^*−/−*^ skulls compared to wild-type skulls (Supplementary Fig. 5a). This affected the mandible morphology as the angle formed by the two hemi-mandibles was significantly increased in *Slc10a7*^*−/−*^ mice compared with wild-type littermates (Supplementary Fig. 5b). Out of six analysed *Slc10a7*^*−/−*^ mice, two presented with a deviated nasal bone (Supplementary Fig. 5c) and one with a shortened nasal bone, whereas no nasal bone abnormalities were observed in *Slc10a7*^*+/−*^ and *Slc10a7*^*+/+*^ mice, suggesting an incomplete penetrance for that phenotype. No significant differences were observed in any morphometric measurements performed comparing *Slc10a7*^*+/−*^ and *Slc10a7*^*+/+*^ mice.

### *SLC10A7* deficiency leads to craniofacial and tooth anomalies

**Fig. 4:**
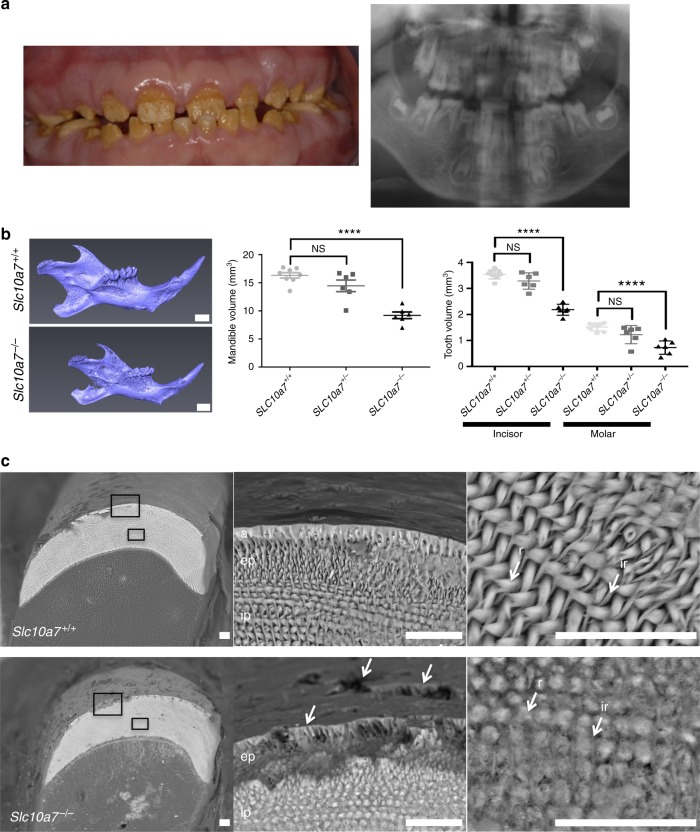
*SLC10A7* deficiency leads to enamel anomalies in human and in mice. **a** Intra-oral photography of Patient 4 at 9 years of age showing hypomineralized amelogenesis imperfecta (left panel). X-ray panoramic of Patient 5 at 6 years of age showing absence of enamel radiolucency corresponding to amelogenesis imperfecta associated with severe oligodontia (right panel). **b** Three-dimensional reconstruction of mandibles from μCT analysis of 8-week-old mouse skulls and volume measurement of mandibles, lower incisors and lower molars at 8 weeks. Scale bars = 1 mm. Data are expressed as mean ± SD. NS, nonsignificant; ****p ≤ 0.0001 (two-tailed *t-*test). *n* = 7 (*Slc10a7*^*+/+*^), *n* = 7 (*Slc10a7*^*+/−*^) and *n* = 6 (*Slc10a7*^*−/−*^). **c** Scanning electron microscopy of mandible incisor from *Slc10a7*^*+/+*^ and *Slc10a7*^*−/−*^ mice. Low magnification (left panels) shows conservation of enamel morphology but decreased thickness in *Slc10a7*^*−/−*^ mice. The boxed areas in the left panels are shown at higher magnification (middle and right panels). In *Slc10a7*^*−/−*^ mouse enamel, the aprismatic layer was absent and the external prismatic layer was altered giving a rough aspect to the enamel surface (middle panels: arrows indicate hole in the external prismatic layer; a = aprismatic enamel layer, ep = external prismatic layer, ip = internal prismatic layer). High magnification of internal prismatic enamel shows absence of a well-defined prismatic pattern in *Slc10a7*^*−/−*^ mice, with fused rods and inter-rod structures (right panels; r = rod, ir = inter-rod). Scale bars = 20 μm. These images represent three incisors analysed

As described earlier, all six patients were diagnosed with hypomineralized amelogenesis imperfecta. More precisely, intra-oral examination revealed yellow–brown enamel with a rough surface. Tooth crowns were short and widely spaced giving the appearance of mild microdontia. The enamel layer could not be distinguished on the panoramic radiography, indicating hypomineralization of enamel (Fig. 4a) and tooth agenesis was observed. To determine whether *Slc10a7* deficiency also results in tooth defects in mice, mandibles and teeth of *Slc10a7*^*−/−*^ mice were analysed. Three-dimensional analyses of mouse heads revealed a decrease in volume of all anatomical structures (mandible, molars and incisors) in the same proportion (Fig. 4b). Mature enamel from the incisors was analysed by scanning electronic microscopy. Enamel thickness was not proportionally decreased in *Slc10a7*^*−/−*^ mice compared with wild-type littermates as the ratio of enamel thickness to incisor thickness was similar in *Slc10a7*^*−/−*^ mice and wild-type littermates, and incisor morphology was conserved (Supplementary Fig. 6). In wild-type mice, enamel consists of three layers: aprismatic (without enamel rods), external prismatic (all prisms are cut transversally) and internal prismatic (prisms are alternatively cut in sagittal and transverse orientation). In the *Slc10a7*^*−/−*^ mouse incisors, the most external layer, the aprismatic enamel layer, was missing (Fig. 4c). Furthermore, numerous areas of hypoplasia were observed in the external prismatic enamel layer of *Slc10a7*^*−/−*^ mice (Fig. 4c). High magnification of the prismatic enamel layer in S*lc10a7*^*−*/*−*^ mice revealed less well-defined rods and inter-rod structures compared with wild-type mice (Fig. 4c).

### *Slc10a7* deficiency alters long-bone microarchitecture

**Fig. 5:**
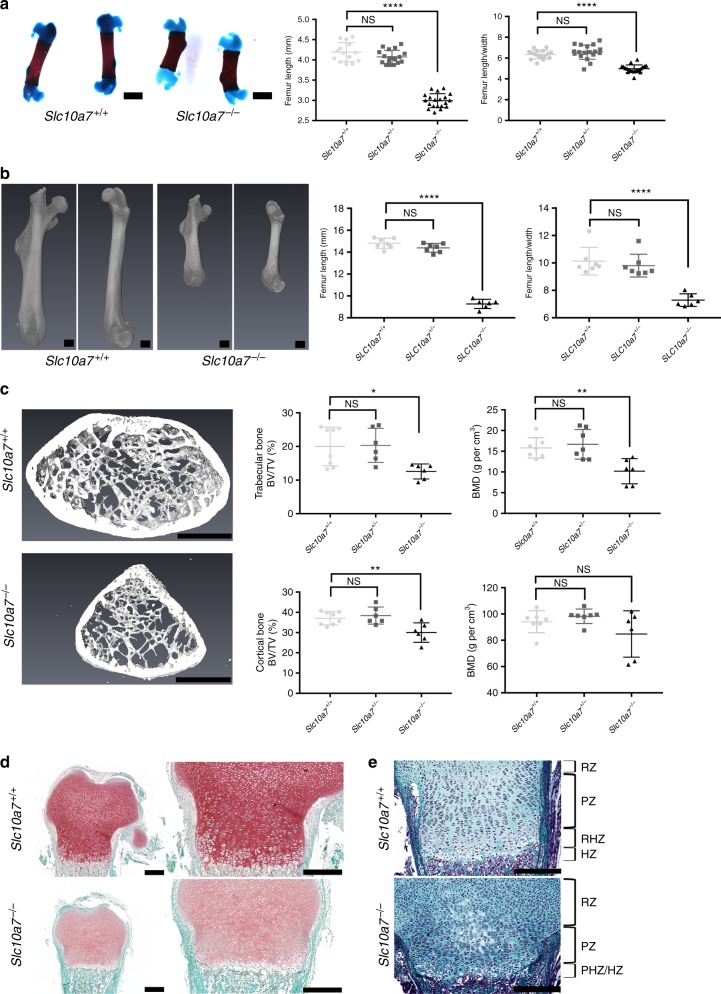
*Slc10a7*^*−/−*^ mice exhibit long-bone macro- and microstructure defects. **a** Alizarin red/Alcian blue staining of newborn femurs and measurement of newborn femur length and femur length/width ratio. Scale bars = 1 mm. *n* = 8 (*Slc10a7*^*+/+*^), *n* = 9 (*Slc10a7*^*+/−*^) and *n* = 10 (*Slc10a7*^*−/−*^). **b** Three-dimensional reconstruction of μCT analysis of 8-week-old mouse femurs and measurement of 8-week-old femur length and femur length/width ratio. Scale bars = 1 mm. Panels (**a**) and (**b**) demonstrate that *Slc10a7*^*−/−*^ femurs, both at birth and at 8 weeks, are shorter and thicker, and exhibit morphological defects. **c** Three-dimensional μCT of sections of 8-week-old distal femur metaphyses from *Slc10a7*^*+/+*^ and *Slc10a7*^*−/−*^ mice. Scale bars = 1 mm. Graphs show trabecular and cortical bone volume (BV/TV) and bone mineral density (BMD). *n* = 7 (*Slc10a7*^*+/+*^), *n* = 7 (*Slc10a7*^*+/−*^) and *n* = 6 *Slc10a7*^*−/−*^). **d** Safranin O staining of the distal femur epiphysis of newborn *Slc10a7*^*+/+*^ and *Slc10a7*^*−/−*^ mice. Right panels show higher magnification of the growth plate. Scale bars = 250 μm. **e** Masson’s Trichome staining of distal femur epiphysis of newborn *Slc10a7*^*+/+*^ and *Slc10a7*^*−/−*^ mice. Scale bars = 250 μm. HZ, hypertrophic zone; PHZ, prehypertrophic zone; PZ, proliferative zone; RZ, resting zone. Images are representative of *n* = 10 and *n* = 5 per group for Safranin O and Masson’s Trichome staining, respectively. Data are expressed as mean ± SD. NS, nonsignificant; *p ≤ 0.05; **p ≤ 0.0.1; ****p ≤ 0.0001 (two-tailed *t-*test)

Both at birth and at 8 weeks, long bones of *Slc10a7*^*−/−*^ mice were shorter and thicker than wild-type mouse bones, as demonstrated by the reduced length/width ratio (Fig. 5a, b). At 8 weeks, *Slc10a7*^*−/−*^ femurs exhibited enlarged distal condyles, more prominent proximal trochanters and shorter necks, giving the specific “Swedish key” aspect to the proximal femur observed on the X-rays of *SLC10A7*-deficient patients (Fig. 5b). To examine whether the morphological alterations of *Slc10a7*^*−/−*^ mouse femurs were associated with microstructural bone defects, μCT analyses were performed on femur distal condyles from 8-week-old mice. Morphological defects were detected in the three-dimensional reconstructions of femur metaphysis sections of *Slc10a7*^*−/−*^ mice, where a triangular shape instead of the elliptic shape seen in wild-type bones was observed (Fig. 5c). Quantitative analyses revealed that *Slc10a7*^*−/−*^ mice have a significantly lower bone volume/total volume ratio, both for trabecular bone and cortical bones compared with wild-type littermates (Fig. 5c). Trabecular thickness (Tb.Th.) but not trabecular number (Tb.N.) was significantly reduced (Tb.Th.: 0.0525 mm ± 0.0053 [*Slc10a7*^*+/+*^] vs. 0.0399 mm ± 0.0021 [*Slc10a7*^*−/−*^], *p* < 0.001; Tb.N.: 3.76 mm^−1^ ± 0.825 [*Slc10a7*^*+/+*^] vs. 3.14 mm^−1^ ± 0.526 [*Slc10a7*^*−/−*^], *p* = 0.13), and bone mineral density was significantly decreased in trabecular but not cortical bone of *Slc10a7*^*−/−*^ mice compared with wild-type littermates (Fig. 5c).

Safranin O staining of histological sections from newborn distal femurs demonstrated a reduction in the size of epiphyses in *Slc10a7*^*−/−*^ mice compared with wild-type mice. There was a strong reduction of Safranin O staining, a marker of sulfated GAG chains, in *Slc10a7*^*−/−*^ mice compared with wild-type mice, which was associated with disorganization of the growth plate (Fig. 5d). This disorganization was more visible when histological sections were stained with Masson’s Trichrome. Overall, the *Slc10a7*^*−/−*^ growth plates were much thinner than wild-type growth plates. The different chondrocyte layers, i.e., resting, proliferative, prehypertrophic and hypertrophic zones, were less discernible in *Slc10a7*^*−/−*^ mice compared with the wild-type littermates (Fig. 5e). The columnar organization of proliferative chondrocytes was visible; however, proliferative chondrocytes were more tightly packed and the thickness of the proliferative zone was reduced in *Slc10a7*^*−/−*^ mice. The prehypertrophic/hypertrophic zone was the most affected layer in *Slc10a7*^*−/−*^ growth plates, the thickness of the hypertrophic zone in *Slc10a7*^*−/−*^ mice was limited to two to three cells with anarchic alignment. Interestingly, a denser blue staining (corresponding to collagen fibres) was observed in the growth plates of *Slc10a7*^*−/−*^ mice compared with wild type. Together, these results suggest an alteration in the composition of extracellular matrix, possibly due to a reduced proteoglycan/collagen ratio leading to growth plate disorganization and bone growth delay.

### SLC10A7 deficiency leads to GAG biosynthesis defect

**Fig. 6:**
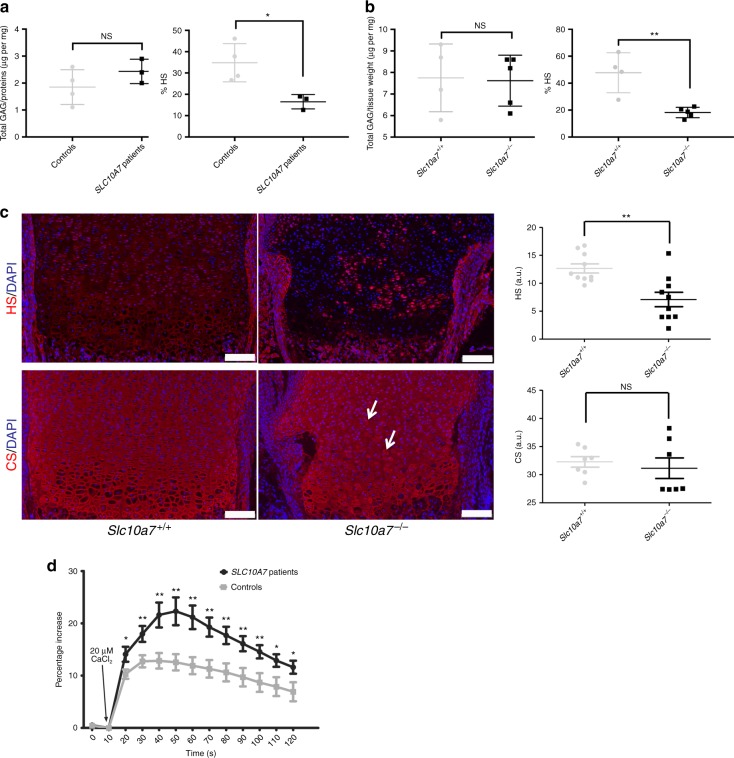
*SLC10A7* deficiency leads to defective GAG and enhanced Ca^2+^ intake. **a**, **b** Total sulfated GAGs and heparan sulfates (HS) were quantified according to the DMMB procedure in extracts of *SLC10A7*-deficient patient fibroblasts and control fibroblasts (*n* = 3) (**a**) or in cartilage extracts from 10-day-old *Slc10a7*^*−/−*^ or *Slc10a7*^*+/+*^ mice (*n* = 5) (**b**). Proportions of HS are expressed as a percentage of total sulfated GAGs (% HS). **c** Immunohistofluorescence for HS (red) or CS (red) counterstained with DAPI (blue) on distal femurs of newborn *Slc10a7*^*+/+*^ and *Slc10a7*^*−/−*^ mice (*n* = 5 mice). Arrows indicate more intense CS staining at the close proximity of chondrocytes. Scale bars = 100 μm. Graphs show red fluorescent signal intensity in the growth plate for each marker. a.u., arbitrary unit. Data are expressed as mean ± SD. NS, nonsignificant; **p ≤ 0.01 (two-tailed *t-*test). **d** A representative recording of intracellular free Ca^2+^ in *SLC10A7*-deficient patients fibroblasts and control fibroblasts (*n* = 3). Fibroblasts were loaded with Fluo-4-AM and preincubated in calcium-free buffer for 30 min before addition of 20 μM CaCl_2_. Data are presented as mean ± SEM, *p ≤ 0.05; **p ≤ 0.0.1 (two-tailed *t-*test)

To confirm the histological analyses suggesting a proteoglycan deficiency in *Slc10a7*^*−/−*^ growth plates, GAG content from *SLC10A7*-deficient patient fibroblasts and in cartilage extracts from 10-day-old *Slc10a7*^*−/−*^ mice was measured. Although no significant difference in total GAG was detected in either sample compared with controls (Fig. 6a, b), the proportion of heparan sulfate (HS) was significantly reduced by ~2-fold in *SLC10A7*-deficient patient fibroblasts compared with control fibroblasts and by about 2.5-fold in *Slc10a7*^*−/−*^ mouse cartilage compared with wild-type mouse cartilage (Fig. 6a, b). Interestingly, no significant difference was evident in the proportion of HS in muscle (measured as a control tissue), between *Slc10a7*^*−/−*^ mice and *Slc10a7*^*+/+*^ mice (Supplementary Fig. 7a). To investigate whether the HS from *Slc10a7*^*−/−*^ mouse cartilage were altered in their sulfation partners, we digested them with a heparitinase cocktail to generate HS-derived sulfated disaccharides that could reflect altered sulfotransferases activities. High-performance liquid chromatography (HPLC) analysis of disaccharides from *Slc10a7*^*−/−*^ and *Slc10a7*^*+/+*^ mice showed no difference in their sulfation patterns (Supplementary Fig. 7b), indicating non-altered HS sulfotransferases activities in the *Slc10a7*^*−/−*^ mice. We then examined the GAGs chain length by gel electrophoresis and, again, non-difference was detected in HS chain size between *Slc10a7*^*−/−*^ and *Slc10a7*^*+/+*^ mice (Supplementary Fig. 7c), indicating that HS decrease in *Slc10a7*^*−/−*^ is mostly related to the number of HS chains, rather than to their sulfation and length.

Immunohistofluorescence experiments were used to visualize HS and chondroitin sulfate (CS) in newborn mouse cartilage. From birth, the overall intensity of HS immunostaining was significantly reduced in *Slc10a7*^*−/−*^ mice compared with wild-type mice (Fig. 6c). However, the distribution of HS immunostaining in *Slc10a7*^*−/−*^ cartilage was variable with markedly reduced HS immunostaining of extracellular matrix being accompanied by intensely stained chondrocytes. No significant difference in CS immunostaining intensity was measured between *Slc10a7*^*−/−*^ growth plates and wild-type controls. However, CS immunostaining was less homogenous with more intense staining at close proximity to some chondrocytes in *Slc10a7*^*−/−*^ growth plates compared with wild-type controls (Fig. 6c). These data provide further evidence that *SLC10A7* deficiency results in impaired GAG biosynthesis.

Based on the fact that SLC10A7 yeast orthologs are known to be putative negative regulators of cytosolic calcium homoeostasis^15,16^, free intracellular calcium in *SLC10A7-*deficient patient fibroblasts and control fibroblasts was assessed. After addition of extracellular CaCl_2_, *SLC10A7*-deficient patient fibroblasts showed a significantly increased Ca^2+^ influx compared with control fibroblasts (Fig. 6d).

### *SLC10A7* deficiency leads to altered *N*-glycoprotein pattern

Finally, the electrophoretic profile of *N*-glycoproteins, orosomucoid, haptoglobin, transferrin and alpha-1-anti-trypsin, was analysed from dried blood spots obtained from *SLC10A7* patients in order to determine whether these proteins carried *N*-glycosylation defects. By classical SDS-polyacrylamide gel electrophoresis (PAGE), orosomucoid and haptoglobin migrated faster in the patient samples than in the control samples (Supplementary Fig. 8). Two-dimensional electrophoresis showed a shift of the far right haptoglobin glycoforms. Together, these results suggest an impact on remodelling of glycans, resulting in truncated and abnormal glycan structures.

## Discussion

This study presents evidence that homozygous mutations in *SLC10A7* are responsible for skeletal dysplasia and amelogenesis imperfecta. Five *SLC10A7* variants were identified in four patients from four unrelated families and two patients from two distantly related families, segregating according to a recessive mode of inheritance (Supplementary Fig. 1b). *SLC10A7* mutations were shown to impair SLC10A7 protein synthesis, as evidenced by the reduced signal detected by immunocytofluorescence and western blot analyses. Inactivation of *Slc10a7* in a mouse model resulted in a complex phenotype, including abnormal development of skeletal structures and teeth anomalies that recapitulated the human phenotype, supporting the conclusion that *SLC10A7* mutations are the cause of the clinical phenotype.

Individuals with *SLC10A7* mutations presented with severe pre- and postnatal disproportionate short stature, microretrognathia, dislocations with monkey wrench appearance of the femora, short long bones with metaphyseal widening and advanced carpal and tarsal ossification. Of particular interest, all patients were diagnosed with amelogenesis imperfecta, while no tooth anomalies have been described in the group of skeletal dysplasias with multiple dislocations^2,6,7^. Thus, amelogenesis imperfecta can be considered as a new clinical feature indicative of *SLC10A7* mutations.

SLC10A7, previously named C4orf13^17^, is a 340-amino acid 10-transmembrane-domain protein localized at the plasma membrane (confirmed by the immunocytofluorescence assay performed in this study). It is a member of the SLC10 family that comprises a Na^+^/taurocholate co-transporting polypeptide (SLC10A1) and an apical sodium-dependent bile acid transporter (SLC10A2). SLC10A7 is an atypical member of this carrier family both with regards to its genomic organization, membrane topology and transport function. The *SLC10A7* gene is composed of 12 exons and is present in vertebrates, plants, yeast and bacteria^14^. Its specific function and substrate remain unknown. For example, no transport activity was detected for the bile acids taurocholate, cholate and chenodeoxycholate, and the steroidsulfates estrone-3-sulfate, dehydroepiandrosterone sulfate and pregnenolone sulfate^14^. However, two studies performed with SLC10A7 homologues in yeast, CaRch1p and Rch1p, suggest a possible role as a negative regulator of cytosolic calcium homoeostasis^15,16^. Furthermore, a study on high-throughput screening of mouse gene knockouts very succinctly described a moderate skeletal dysplasia associated with loose joints in a *Slc10a7*^*−/−*^ mouse model^18^

In situ hybridization confirmed the broad expression of *Slc10a7* mRNA expression in mouse as previously described^14,17^. *SLC10A7* mRNA was more specifically expressed in tissues affected in patients, i.e., cartilage giving rise to long bones and long-bone growth plates (skeletal dysplasia), emerging teeth (amelogenesis imperfecta), lungs and developing heart (congenital heart defect in one patient), strengthening the implication of SLC10A7 deficiency in the occurrence of those clinical features.

A *Slc10a7*-deficient mouse model was generated to further understand the function of SLC10A7. The choice of a constitutive knock-out mouse model was coherent with the loss of function mutations identified in patients. Even though a Slc10a7^*−*/*−*^ mouse model was previously described^18^, it only provided a brief analysis of long bones and joint in adult mouse. Our *Slc10a7*^*−/−*^ mice presented with skeletal dysplasia including growth retardation at birth and at 8 weeks, alteration of long-bone morphology, craniofacial anomalies and advanced tarsal maturation at birth, associated with enamel defects, demonstrating a strong correlation with the human phenotype. However, no obvious joint dislocation was observed in *Slc10a7*^*−/−*^ mice. This may mean that *Slc10a7*^*−/−*^ mice differ from patients and do not have joint abnormalities. More likely, more subtle joint defects are present and that joint laxity is only detectable by specific measurement,^19^ or joint abnormalities would appear in older mice as loose joints were described in another *Slc10a*7^*−*/*−*^ mouse model^18^.

Immunohistofluorescent analyses of newborn mouse growth plates and GAG quantification in patient fibroblasts and in mouse cartilage all demonstrated an alteration of proteoglycan GAG moieties synthesis, a phenotype previously described for other chondrodysplasias with multiple dislocations^2,6,7,13^. Proteoglycans are extracellular matrix macromolecules where GAG chains, consisting of repeating sulfated disaccharide units, are attached to a core protein. The cellular functions of these proteoglycans are fundamental and mainly depend on the composition of the GAGs. The GAG biosynthesis, initiated at the exit of endoplasmic reticulum, takes place essentially in the Golgi apparatus^20^. In the secretory pathway, normal glycosylation, and thus GAG biosynthesis, is highly dependent on a correct Golgi cisternae organization, with shallow pH gradient and regulated enzymatic and ionic content^12,21^. For example, Ca^2+^concentration has a major role in that process as calcium depletion from Golgi and endoplasmic reticulum lumens induced by thapsigargin affects collagen and proteoglycans synthesis^22^, and increased extracellular Ca^2+^ levels also reduces proteoglycan secretion^23,24^. In this study, *SLC10A7* deficiency was demonstrated to lead to a proteoglycan synthesis defect and, more specifically, to decreased HS content. HS biosynthesis is initiated by the addition of *N*-acetylglucosamine (GlcNac), catalysed by EXTL2 and EXTL3 enzymes. EXT1 and EXT2 are then responsible for the chain elongation by adding alternating glucuronic acid and GlcNac sugar subunits. HS final structure and specific function is finally modulated by epimerisation, sulfation and deacetylation^25^. The unchanged HS chain length and sulfation pattern in the *Slc10a7*^*−*/*−*^ deficient mouse cartilage indicates that the decreased HS levels are associated to a reduced number of HS chains. These findings suggest an impairment of the initial step of HS chain synthesis. This significant reduction in HS, while the total GAG concentration was not altered, could be explained by compensatory CS synthesis, as observed in *Ext1*^*−/−*^ ES cells^26^ and *EXTL3*-deficient patients^27^. In addition to altered proteoglycan synthesis, an abnormal electrophoretic pattern for two *N*-glycoproteins, oromucosoid and haptoglobin, was observed, suggesting that in addition to GAG synthesis SLC10A7 deficiency may also affect other types of glycosylation^28^.

Very interestingly, *SLC10A7* deficiency was also responsible for an increased Ca^2+^ intracellular intake in skin fibroblasts, confirming a role for SLC10A7 in intracellular calcium homoeostasis, as previously suggested by studies in yeast homologues. This deregulation of Ca^2+^ homoeostasis due to *SLC10A7* deficiency is most likely responsible for defects in GAG synthesis and glycosylation.

Bone formation and development, through endochondral ossification, are governed by gradients of signalling molecules, including Indian hedgehog (Ihh), parathyroid hormone-related protein, fibroblast growth factors, Wnt proteins and bone morphogenic proteins^29^. Proteoglycans, such as CS proteoglycans (CSPGs) and HS proteoglycans (HSPGs), are important modulators of signalling modulator gradients. Several studies have shown that CSPGs and HSPGs have different effects, and in some cases opposite effects, on the regulation of growth factor-mediated signal transductions, as demonstrated, e.g., for Ihh diffusion^30,31^. Thus, an alteration of the CSPGs/HSPGs ratio, as observed in our study, could affect the signalling pathways regulating chondrocyte proliferation and differentiation, explaining the growth plate disorganization observed in *Slc10a7*^*−/−*^ mice. Although phenotypic similarities between the *pug* mutant, i.e., *Xylt1*-deficient mice, and our *Slc10a7*^*−/−*^ mouse model suggest that the underlying mechanisms leading to growth defects are similar, i.e., premature chondrocyte maturation and early ossification^32^, further analyses of growth factor signalling pathways are necessary to fully understand the pathogenesis of the skeletal dysplasia induced by *SLC10A7* deficiency.

Finally, all individuals with *SLC10A7* mutations presented with a hypomineralized amelogenesis imperfecta. Analyses of *Slc10a7*^*−/−*^ mouse teeth demonstrated a defective outermost enamel layer and less clearly defined enamel rods, whereas the global enamel thickness was not affected, suggesting alterations in enamel maturation and/or mineralization. Mature enamel is the hardest and the most mineralized tissue in the human body. Amelogenesis, or enamel formation, is orchestrated by ameloblasts, which are responsible for the synthesis of the protein matrix scaffold and the calcium hydroxyapatite crystal deposition^33^. Although no proteoglycan defects have been associated with amelogenesis imperfecta in humans, several mouse studies have demonstrated the implication of proteoglycans, such as perlecan and decorin, in enamel formation^34,35^. As mentioned above, amelogenesis imperfecta seems to be a phenotypic feature specific to *SLC10A7* deficiency within the group of chondrodysplasias with multiple dislocations, suggesting that amelogenesis imperfecta is not due to a defect in proteoglycan synthesis but to a different function of SLC10A7. It can rather be explained by a role of SLC10A7 in Ca^2+^ influx homoeostasis suggested by the increased Ca^2+^ intracellular intake in SLC10A7-deficient patients fibroblasts. Indeed, during the maturation stage, which seems to be affected by SLC10A7 deficiency, an increase in active transport of calcium by the ameloblasts in the enamel occurs. Furthermore, mutations in genes encoding for Ca^2+^ transporters, such as SLC24A4, have been identified in patients with amelogenesis imperfecta^36^.

In conclusion, our functional work-up of *SLC10A7* has identified a new gene responsible for skeletal dysplasia and amelogenesis imperfecta, illustrating the complexity of GAG synthesis and the putative role of Ca^2+^ homoeostasis in this process, thus opening new possibilities for the development of therapeutic approaches by correcting the defective Ca^2+^ homoeostasis in the Golgi.

## Methods

### Affected individuals

The affected individuals selected for this study fulfilled the diagnostic criteria for chondrodysplasia with multiple dislocations, namely short stature and dislocations of large joints. GeneMatcher^37^ was used to identify other physicians caring for patients with variants in *SLC10A7*.

### Samples

The studies were approved by the ethics committees of the Necker Hospital (Paris) and Julius Maximilians University Würzburg. Parents or guardians provided written informed consent for the biochemical and genetic analysis and the publication of photographs and clinical data. The authors affirm that human research participants provided informed consent for publication of the images in Fig. 1. DNA was extracted from venous blood using QIAamp DNA blood Maxi kit (QIAGEN). Fibroblast cultures were established from skin biopsies.

### Exome sequencing

Exome capture was performed at the genomic platform of the IMAGINE Institute (Paris, France) with the SureSelect Human All Exon kit (Agilent Technologies) for the three Turkish patients, at the University Medical Center Utrecht and University Medical Center Groningen for the two Dutch patients and at the Institute of Human Genetics at the Julius Maximilians University Würzburg for the Iranian patients. Agilent SureSelect Human All Exon (V4) libraries were prepared from 3 μg of genomic DNA sheared with ultrasonicator (Covaris) as recommended by the manufacturer.

Barcoded exome libraries were pooled and sequenced using a HiSeq2500 (Illumina), generating paired-end reads. After demultiplexing, sequences were mapped to the human genome reference (NCBI build37/hg 19 version) with Burrows–Wheeler Aligner^38^. The mean depth of coverage obtained for each sample was ≥ × 80 with 95% of the exome covered at least × 15. Variant calling was carried out with the Genome Analysis Toolkit (GATK)^39^, Sequence Alignment/Map tools^40^ and Picard Tools. Single-nucleotide variants were called with GATK Unified Genotyper, whereas indel calls were made with the GATK IndelGenotyper_v2. All variants with a read coverage ≤ × 2 and a Phred-scaled quality of ≤ 20 were filtered out. Variants were annotated and filtered using an in-house annotation software system (Polyweb, unpublished).

The analyses focused on non-synonymous variants, splice variants, and coding indels. Variant pathogenicity was evaluated using the prediction algorithms SIFT (cutoff ≤ 0.05), PolyPhen-2 (HumVar scores, cutoff ≥ 0.447) and Mutation Taster (cutoff: qualitative prediction as pathogenic). The variant frequency in control datasets was assessed, including the ExAC database, dbSNP129, the 1000 Genomes project, ClinVar, HGMD and in-house exome data. All variants were confirmed by Sanger sequencing and segregation was verified.

### Sequencing analysis of *SLC10A7*

The exons and exon–intron boundaries of *SLC10A7* were amplified with specific primers (Supplementary Table 1). Amplification products were purified by ExoSapIT (Amersham) and directly sequenced with the Big Dye Terminator Cycle Sequencing Ready Reaction kit v1.1 on an automatic sequencer (3500XL and 3130XL; PE Applied Biosystems). Sequence analyses were performed with the analysis software, Sequencing 6 (Applied Biosystems) and Gensearch (PhenoSystems SA).

### SLC10A7 expression plasmids

Skin primary fibroblasts (control and case 1 and 3) were cultured in RPMI medium supplemented with 10% fetal calf serum. Total RNAs from fibroblast monolayers were extracted using the RNeasy Mini kit (Qiagen) according to the manufacturer’s instructions. *SLC10A7* cDNA was amplified after reverse transcription of RNA using the forward primer 5′-AAGGATCCCCCTAACAAATATGAGGCTGCTGG-3′ (BamHI restriction site underlined) and the reverse primer 5′-AACTCGAGGTATACTGTCGGCCTTGTCAGCTT-3′ (XhoI restriction site underlined). The resulting amplicons were cloned into pcDNA™3.1/*Myc*-His A + (Invitrogen) to generate proteins with an in-frame *Myc*-His Tag and then sequenced to verify the correct insertion.

### Recombinant protein expression

HEK293F cells, COS-1 cells and MG63 cells (ATCC) were cultured in Dulbecco’s modified Eagle’s mmedium supplemented with 10% fetal bovine serum (FBS). Transfections were performed on cells in 24-well plates or in 8-chamber labtek slides (ThermoFisher Scientific) using jetPRIME® transfection reagent (Polyplus Transfection) according to the manufacturer’s instructions.

For western blotting, cells in 24-well plates were collected 48 h after transfection and lysed in denaturation buffer. Polyacrylamide gel electrophoresis, transfer and immunoblotting were performed according to standard protocols using monoclonal anti-myc (9E10; 1/1000; Santa Cruz Biotechnologies) or monoclonal anti-actin (clone C4; 1/5000; Millipore) primary antibodies and goat anti-mouse HRP-conjugated secondary antibody (1/2000; Novex, Life Technologies, catalogue number A16066).

For immunofluorescence, cells in 8-chamber slides were fixed 48 h after transfection with 4% paraformaldehyde (PFA) at room temperature for 30 min. The washed cell layer was incubated sequentially in phosphate-buffered saline (PBS) containing 1% bovine serum albumin (BSA) for 30 min, mouse monoclonal anti-myc antibody for 1 h and Alexa Fluor 594 goat anti-mouse IgG (1/200; Life Technologies, catalogue number A11005) for 1 h. After mounting in Prolong gold antifade mountant with DAPI (Molecular Probes, Life Technologies) cells were observed with an Axioplan2 imaging microscope (Zeiss).

### In situ hybridization

Probes for *SLC10A7* and *Slc10a7* corresponded to nucleotides 46-948 of GenBank accession NM_001029998.5 and nucleotides 429-717 of GenBank accession NM_001009981.2, respectively. Synthetic cDNAs (Eurofins Genomics) were used to generate antisense and sense cRNA probes using a SP6/T7 DIG RNA labelling kit (Roche) and digoxigenin-11-UTP (Roche) according to the manufacturer's specifications. Paraffin sections of paraformaldehyde-fixed human fetuses at 6 and 7 weeks of gestation (Carnegie stages 16 and 19, respectively; obtained with institutional review board approval), murine wild-type embryos at embryonic age E12.5, E14.5 and E16.5, and tissues from wild-type newborn and 10-day-old mice were deparaffinized. These sections were treated with 20 μg/ml Proteinase K for 8 min at 37 °C and postfixed with 4% PFA for 12 min. After washing in PBS and 2 × Saline Sodium Citrate Buffer (SSC), they were acetylated in 0.25% acetic anhydride in 0.1 M triethanolamine for 10 min. The sections were hybridised to 5 μg/ml DIG-11-UTP-labelled *SLC10A7* or *Slc10a7* cRNA probes in hybridization buffer (50% formamide, 10 mM Tris-HCl (pH 7.6), 1 mM EDTA (pH 8), 600 mM NaCl, 10% dextran sulfate sodium salt, 1 mg/ml Yeast tRNA and 1 × Denhardt’s solution) overnight at 70 °C. After hybridization, each slide was washed in 2 × SSC containing 50% formamide at 65 °C for 30 min. Each slide was treated with 20 μg/ml RNAse A in TNE (10 mM Tris-HCl (pH 7.6), 1 mM EDTA, 50 mM NaCl) at 37 °C for 30 min and washed in TNE. Subsequently, the slides were washed twice with 2 × SSC and 0.1  × SSC at room temperature for 15 min each [41]. After staining with 5-bromo-4-chloro-3'-indoylphosphate *p*-toluidine salt and nitroblue tetrazolium chloride in the dark (Roche) according to manufacturer’s recommendations, slides were scanned using a Nanozoomer 2.0 scanner (Hamamatsu) and visualized using NDPviewer (Hamamatsu).

### GAG assays

For analysis of GAG content in primary fibroblasts from patients and controls, 7 × 10^6^ cells at confluence in 75 cm^2^ flasks were washed twice in PBS before treatment with trypsin. Then, RPMI medium was added and cells were centrifuged at 2000 × *g* for 5 min and suspended in 1600 µl of extraction buffer (50 mM Tris-HCl pH 7.9, 10 mM NaCl, 3 mM MgCl_2_ and 1% Triton X-100). After incubation for 15 min at 4 °C, 10 μl was removed for protein quantification with BCA Assay (Thermofisher). Proteins were digested by proteinase K treatment (200 µg/mL) at 56 °C overnight followed by 30 min at 90 °C for inactivation of proteinase K. After cooling, 7.5 U of DNAse I (QIAGEN) was added and the samples incubated overnight at 37 °C. To ensure complete DNA elimination, samples were centrifuged 10 min at 10,000 × *g* in Nanosep MF 0.2 µm tubes from Pall Corporation (France). To complete the separation of protein from GAGs, NaCl was added at a final concentration of 4 M and samples were vortexed for 30 min. Trichloroacetic acid (10% final concentration) was added at 4 °C and samples were stored at this temperature for 15 min. The samples were then centrifuged at 10,000 × *g* for 10 min and the pellet discarded. Trichloroacetic acid (TCA) was eliminated by chloroform extraction followed by serial dialyses of the aqueous phase against a Tris buffer (50 mM Tris-HCl, 50 mM sodium acetate and 2 mM calcium chloride, pH 8) and water. Samples were lyophilized then dissolved in pure water or in glycanase digestion buffer (100 mM sodium acetate, 10 mM calcium chloride, pH 7).

For extraction of GAGs from mouse tissues, cartilages and skeletal muscle from 10-day-old mice were weighed then suspended in 2 ml of extraction buffer and finally their GAGs were extracted as detailed above for cells.

Following extraction, sulfated GAGs were quantified according to the protocol described by Barbosa et al.^42^ with slight modifications. Total sulfated GAGs, amount of HS after chondroitinase treatment (Sigma, 10 U/ml) and of CS after heparitinase I/II/II (0.25 U/ml each) treatment were quantified according to the DMMB (1-9 dimethyl-methylene blue) assay. A calibration curve, constructed with known amounts of a CS-A standard ranging from 0.25 to 3.0 µg, was included in every assay. For the cells, total GAGs quantification is expressed in function of their protein content. For mice cartilage, total GAGs quantification is expressed in function of their dried weight. HS and CS are expressed as percentage of total GAGs. HPLC analysis of HS and GAG length were performed as previously descrided^43,44^.

### Ca^2+^ imaging

Human skin fibroblasts were plated at 15 × 10^3^ cells/cm^2^ in μ-slide 8-well glass bottom (Ibidi) in RPMI media containing 10% FBS. After 24 h, the media was replaced with serum-free fresh media. After 2 h of serum starvation, the cells were incubated in buffer containing fluo-4 dye (2 µM) for 30 min. Cells were washed three times with Ca2 + -free buffer and then kept in Ca^2+^-free buffer (140 mM NaCl, 4 mM KCl, 1 mM MgCl_2_, 25 mM glucose and 10 mM HEPES, pH 7.4). Under fluorescent microscopy (Nikon Instruments Eclipse Ti Inverted Microscopes, NIS-Elements Microscope Imaging software), in Ca^2+^-free buffer, CaCl_2_ (20 mM) was then added extracellularly to facilitate Ca^2+^ influx. Peak fluorescent intensities following CaCl_2_ were captured and average values from at least two sets of cells per patient or control were obtained for analysis. Data were analysed as the percentage of fluorescence intensity before Cacl_2_ for each cell.

### Glycoprotein electrophoretic profile assay

For analysis of glycoproteins from blood spots on Guthrie cards, one circular punched spot was first eluted in 100 μl of distilled water. Blood eluate was fivefold diluted in distilled water. SDS-PAGE was carried out as described by the manufacturer (Life Technologies) using 4–12% NuPAGE Bis-Tris gels and 2 dimensional gel electrophoresis (2-DE) was carried out on 10 μl of blood eluate as described by the manufacturer (Life Technologies) using ZOOM Strip pH 4–7 for the first dimension and 4–12% NuPAGE Bis-Tris gels for the second dimension. In both cases, separated proteins were transferred to nitrocellulose (100 Volts, 1 h) and protein glycoforms haptoglobin (Hpt) and orosomucoid (oroso) were identified using the following rabbit primary antibodies: anti-haptoglobin (Dako, catalogue number A0030; 1/5000 in Tris-Tween-Buffer-Saline); anti-orosomucoid (Dako, catalogue number Q0326; 1/2000 in Tris-Tween-Buffer-Saline (TTBS); anti-transferrin (Siemens, catalogue number OSAX15; 1/4000 in TTBS) and anti-α-anti-trypsin (Siemens, catalogue number OSA209; 1/10,000 in TTBS). HRP-linked anti-rabbit IgG (GE Healthcare, catalogue number NA934V; 1/5000 in TTBS) was used as secondary antibody. Images were acquired using a Chemidoc XRS camera system from Bio-Rad.

### Generation of *Slc10a7*^*−/−*^ mice

All procedures were performed in accordance with the guidelines for animal care of French Animal Care and Use Committee.

*Slc10a7*^*tm1a(EUCOMM)Hmgu*^ ES cells (MGI:1924025) from the EuMMCR were injected into blastocysts from grey C57Bl/6N mice by PolyGene AG. The resulting chimerae were bred with C57Bl/6N mice to obtain *Slc10a7*^*tm1a(EUCOMM)Hmgu/+*^ mice (referred to as *Slc10a7*^*+/−*^). The mice were genotyped using the following primers: 5′-CCGCTTCCTCGTGCTTTAGGTA-3′ and 5′-AACCTCTACAGATGTGATATGGCTG-3′ for transgenic allele amplification, and 5′-GAATCCAGTACAGGAGAGCCACAT-3′ and 5′-TAGAGACCAGGAATTCTGCTAGACA-3′ for wild-type allele amplification. For timed pregnancies, the date of vaginal plug was designated E (embryonic age) 0.5. For all analyses of *Slc10a7*^*+/−*^ and *Slc10a7*^*−/−*^ mice, wild-type littermates were used as controls, and both male and female were used at birth, 10 days of age and 8 weeks of age, as indicated.

### Skeletal staining

Newborn skeletons were skinned, fixed in 95% ethanol, stained with Alcian Blue and Alizarin Red, cleared by KOH treatment and stored in glycerol according to standard protocols.

Images were captured with an Olympus SZX12 stereo-microscope. Bone sizes were measured using ImageJ software.

### X-rays and μCT

Whole mouse skeletons were radiographed using a Faxitron (MX-20). For μCT analyses adult animals were euthanized and femurs and heads were isolated, stripped of soft tissue and stored in 70% ethanol. Three-dimensional microarchitecture of the distal femur was evaluated using a high-resolution Skyscan1076 microtomographic imaging system (Skyscan, Belgium). Images were acquired at 80 KeV, 100 μA, no filter. Three-dimensional reconstructions were generated using NRecon software (Skyscan). Trabecular and cortical measurements were obtained using CTan software (Skyscan) on a set of sections located within the secondary spongiosa under the growth plate and under the secondary spongiosa, respectively. The measured volume was chosen to be proportional to the femur length.

Avizo software (FEI Visualization Sciences Group, Burlington, MA) was used for three-dimensional visualization of heads and for mandible, incisor and molars selection and volume quantification.

### Scanning electron microscopy analysis of incisor enamel

Mandibles from 8-week-old *Slc10a7*^*−/−*^ and *Slc10a7*^*+/+*^ mice (three of each genotype) were dissected out and stored in 70% ethanol. Incisors were cut along the frontal axis at the level of bone emergence using a rotating diamond wheel and optically controlled monitoring. Previous microscanner analysis was used to validate the cutting axis (perpendicular to incisor axis), in order to analyse the buccal part of the incisor. Sample surfaces were polished with sandpaper of successively decreasing grits. Conditioning of the enamel surface was achieved by etching with 37% phosphoric acid for 30 s and carefully drying. Each sample was observed with a scanning electron microscope (TM3030, table top Microscope, Hitachi) at 10 kV. Incisor morphology was used as a criterion for calibration of section planes, based on established μCT-landmarks.

### Histology and immunohistochemistry

Newborn femurs were fixed in 4% paraformaldehyde, decalcified with 0.4 M EDTA before paraffin embedding and 5 μm sections were used for Safranin O and Masson’s trichrome staining or immunofluorescence.

For immunofluorescence, sections were subjected to digestion with 0.5 U/ml chondroitinase ABC (Sigma-Aldrich) for 2 h at 37 °C and, after blocking with 1% BSA, were incubated with anti-HS (1/100, Amsbio, clone F58-10E4, catalogue number 370255-1) and anti-CS (1/200, Amsbio, clone 2B6, catalogue number 270432-CS) antibodies. Alexa fluor 594 goat anti-mouse was used as secondary antibody and slides were mounted in Prolong Gold Antifade with DAPI mounting medium (1/200; Life Technologies, catalogue number A11005) and scanned using a Nanozoomer 2.0 (Hamamatsu). Specific signal intensity was measured with ImageJ software, selecting equivalent areas of the growth plate in each group.

### Statistics

Statistical analyses were performed using GraphPad PRISM. All values are shown as mean ± SD. Statistical differences between two groups were analysed with a two-tailed Student’s *t*-test, assuming a normal distribution. A *p*-value of < 0.05 was considered statistically significant.

### URLs

For 1000 Genomes, see http://www.1000genomes.org. For ExAC Browser, see http://www.exac.broadinstitute.org/. For GeneMatcher, see http://www.genematcher.org. For GenBank, see http://www.ncbi.nim.nih.gov/genbank/. For PolyPhen-2, see http://genetics.bwh.harvard.edu/pph2. For SIFT, see http://www.sift.jcvi.org. For UCSC Genome Browser, see http://www.genome.ucsc.edu. For EUCOMM, see http://www.mousephenotype.org/data/genes/.

### Data availability

The authors declare that all the data supporting the findings of this study are available within the paper and its Supplementary Information files.

## Electronic supplementary material

Suppplementary Information
