# Supplementary material for: SLC10A7 mutations cause a skeletal dysplasia with amelogenesis imperfecta mediated by GAG biosynthesis defects
Source: Nat Commun. 2018 Aug 6;9:3087. doi: 10.1038/s41467-018-05191-8 (PMC6078967; doi:10.1038/s41467-018-05191-8)
Supplement: Supplementary file 1 — Suppplementary Information [file 41467_2018_5191_MOESM1_ESM.pdf]

Supplementary information

SLC10A7 mutations cause a skeletal dysplasia with amelogenesis imperfecta mediated by GAG biosynthesis defects

Dubail *et al.*

a

	Ethnic origin	Consanguinity	Nucleotide change	Status		Location
Family 1	Turkish	Yes	c.774-1G>A	Ho	?	In9
Family 2	Turkish	Yes	c.773+1G>A	Ho	?	In9
Family 3	Turkish	Yes	c.221T>C	Ho	p.Leu74Pro	Ex3
Family 4	Dutch	No	c.514C>T	Ho	p.Gln172*	Ex7
Family 5	Dutch	No	c.514C>T	Ho	p.Gln172*	Ex7
Family 6	Iranian	Yes	c.388G>A	Ho	p.Gly130Arg	Ex4

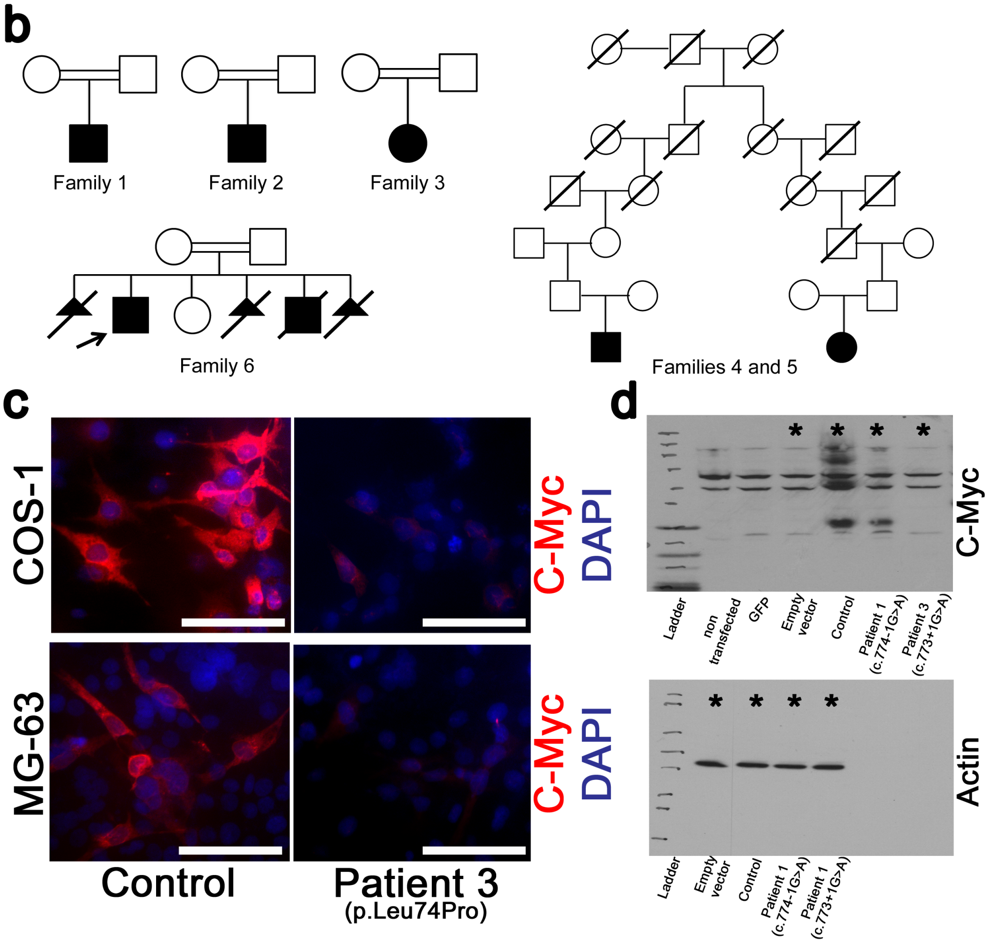

Supplementary Figure 1: ***SLC10A7* mutations and functional consequences of *SLC10A7* mutations**

a) Summary of *SLC10A7* mutations identified in the patients. **b**) Pedigrees of *SLC10A7* patient families. **c)** COS-1 cells and MG63 cells were transfected with plasmids encoding c-myc tagged wild-type or mutant (Patient 3) SLC10A7 protein. Cells were immunostained with anti-c-Myc antibody (red) and nuclei were counterstained with DAPI (blue). Scale bars = 20 µm. The images are representative of 3 independent experiments. **d**) Full unedited western blotting gels for Fig. 2. Western blotting analyses were performed as described in Fig. 2. * indicates the lanes used in Fig. 2.

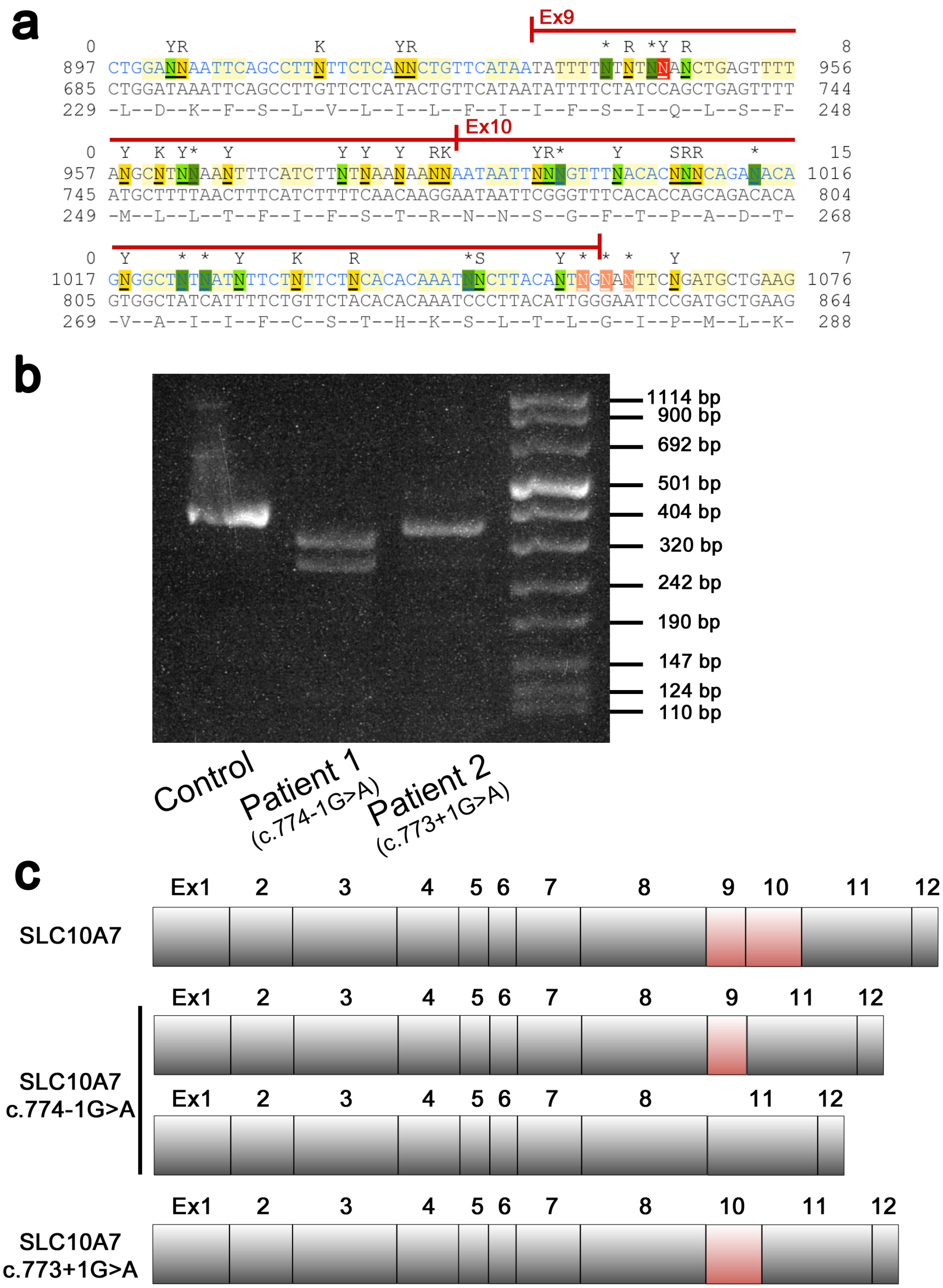

Supplementary Figure 2: **Expression analysis of exon skipping products of *SLC10A7* c.774-1G>A and c.773+1G>A mutants.**

a) Ensembl cDNA and protein sequences of *SLC10A7* exons 9 and 10. **b**) PCR analysis of *SLC10A7* c.774-1G>A and c.773+1G>A transcripts using SLC10A-cDNA-7S and SLC10A7-cDNA-11AS primers (see Supplementary Table 1). **c)** Exon organisation in control and *SLC10A7* c.774-1G>A and c.773+1G>A mutants.

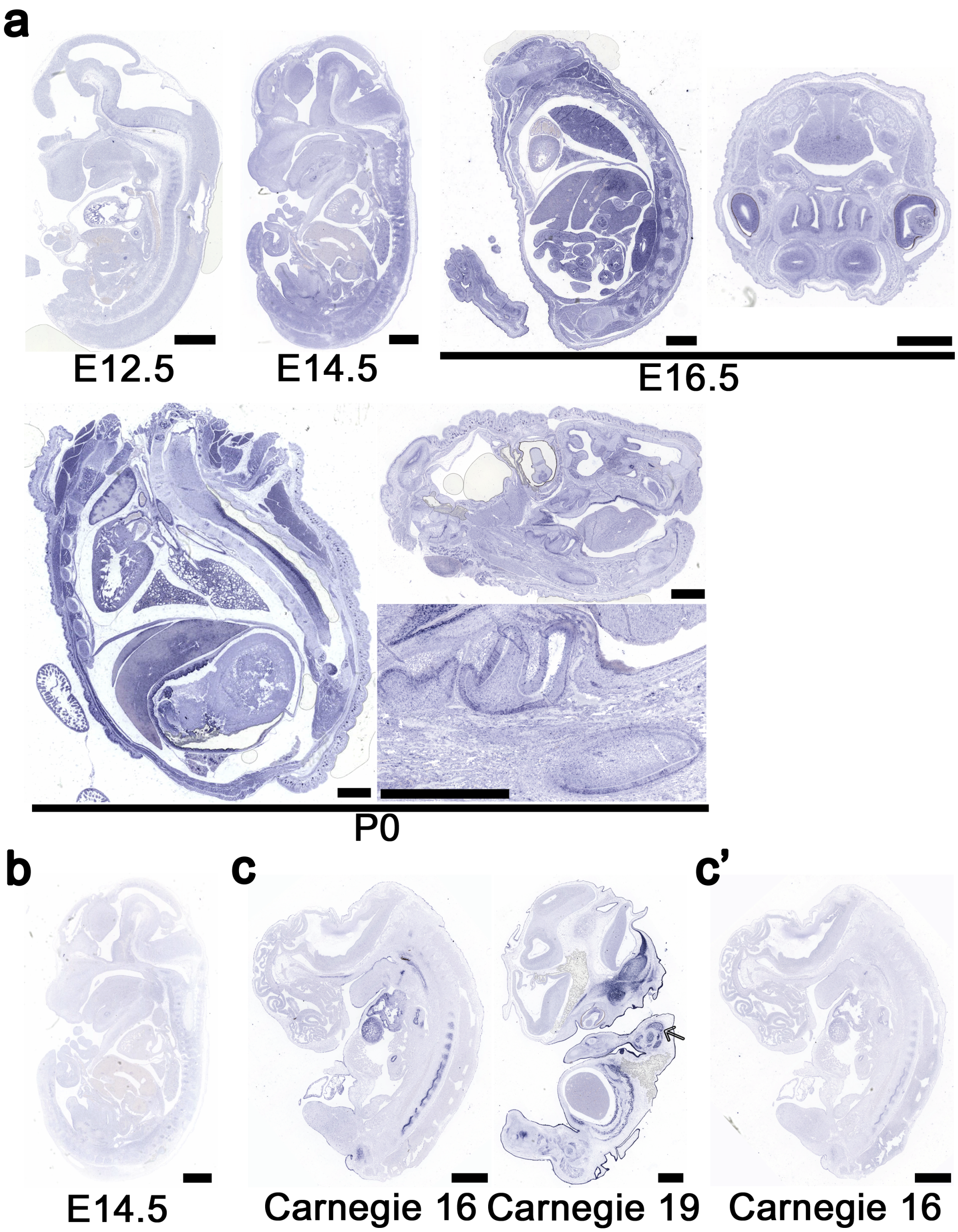

Supplementary Figure 3*: In situ* hybridization analysis of *SLC10A7* mRNA expression in mouse and human embryos.

The blue staining indicates sites of RNA hybridization. **a)** *Slc10a7* mRNA was expressed in mouse embryos from E12.5 to birth (P0). *Slc10a7* mRNA expression in ameloblasts of emerging teeth at birth is shown at higher magnification (lower right panel). **b)** Sense control hybridized to an E14.5 mouse embryo. c) *SLC10A7* mRNA expression in human embryos at 8 weeks (Carnegie stage 16) and 9 weeks (Carnegie stage 19) of gestation. The arrow indicates *SLC10A7* mRNA expression in cartilaginous tissues of the shoulder. **c')** Sense control hybridized to a human embryo at 8 weeks (Carnegie stage 16). Scale bars = 1 mm.

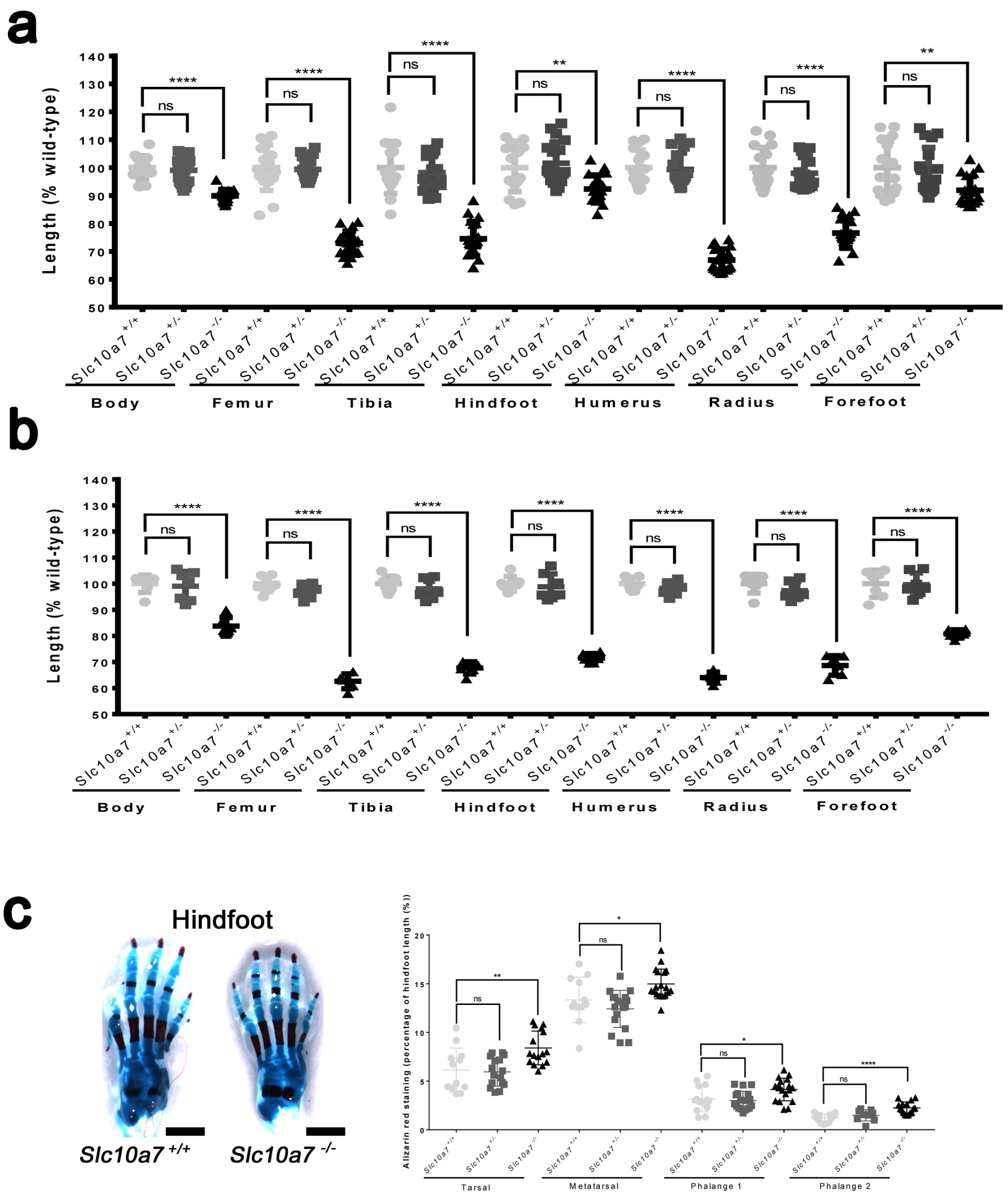

Supplementary Figure 4: ***Slc10a7*$^{-/-}$ mice display skeletal dysplasia associated with advanced hindfoot ossification.**

a, b) Measurement of naso-occipital length and of forelimb and hindlimb bones from newborn **(a)** and 8 week-old mice **(b)**. n = 13 (*Slc10a7*$^{+/+}$), n = 35 (*Slc10a7*$^{+/-}$) and n = 19 (*Slc10a7*$^{-/-}$) at birth and n = 7 (*Slc10a7*$^{+/+}$), n = 7 (*Slc10a7*$^{+/-}$) and n = 6 (*Slc10a7*$^{-/-}$) at 8 weeks. **c)** Alizarin red/Alcian blue preparations of forefeet and hindfeet from newborn mice showing that the ossified regions (stained in red by Alizarin red staining) are more extensive in *Slc10a7*$^{-/-}$ feet compared with *Slc10a7*$^{+/+}$ feet. In the graph, the lengths of ossified regions of different bones in the hindfoot were measured as a percentage of the total hindfoot length. Scale bars = 1 mm. Data are expressed as mean±SD. n.s.: non-significant; *: p≤0.05; **: p≤0.01; ****: p≤0.0001 (two-tailed *t*-test). n = 8 (*Slc10a7*$^{+/+}$), n = 9 (*Slc10a7*$^{+/-}$) and n = 10 (*Slc10a7*$^{-/-}$)

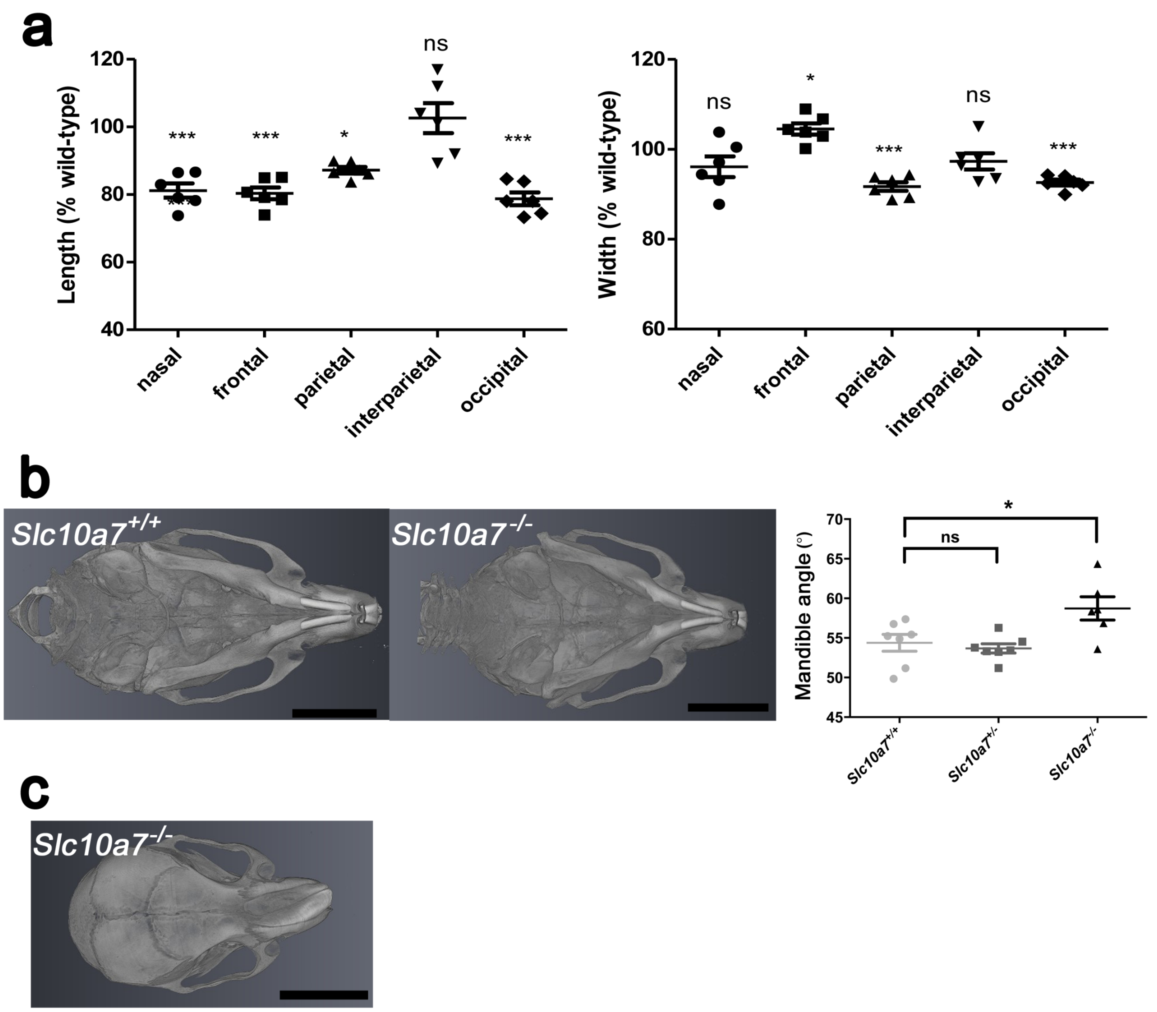

Supplementary Figure 5: *Slc10a7$^{-/-}$* mice display skeletal dysplasia associated with skull anomalies.

a) Lower view of the three-dimensional reconstruction of μCT analysis of 8 week-old mouse skulls exhibiting the increased angle formed by the two hemi-mandibles in *Slc10a7$^{-/-}$* mice compared with wild-type littermates. The graph shows the measurement of the angle formed by the 2 hemi-mandibles. Scale bars = 5 mm. (**b**) skull bone length and width measurements from 8 week-old mice. Data are expressed as mean±SD. n.s.: non-significant; ***: p≤0.001; ****: p≤0.0001 (two-tailed *t*-test). n = 7 (*Slc10a7$^{+/+}$*), n = 7 (*Slc10a7$^{+/-}$*) and n = 6 (*Slc10a7$^{-/-}$*) at 8 weeks. **c)** Three-dimensional reconstruction of μCT analysis of 8 week-old mouse skulls showing the nasal bone deviation observed in 2 *Slc10a7$^{-/-}$* mice out of 6. Scale bars = 5 mm. Data are expressed as mean±SD. n.s.: non-significant; *: p≤0.05; **: p≤0.01; ****: p≤0.0001 (two-tailed *t*-test). n = 7 (*Slc10a7$^{+/+}$*), n = 7 (*Slc10a7$^{+/-}$*) and n = 6 (*Slc10a7$^{-/-}$*).

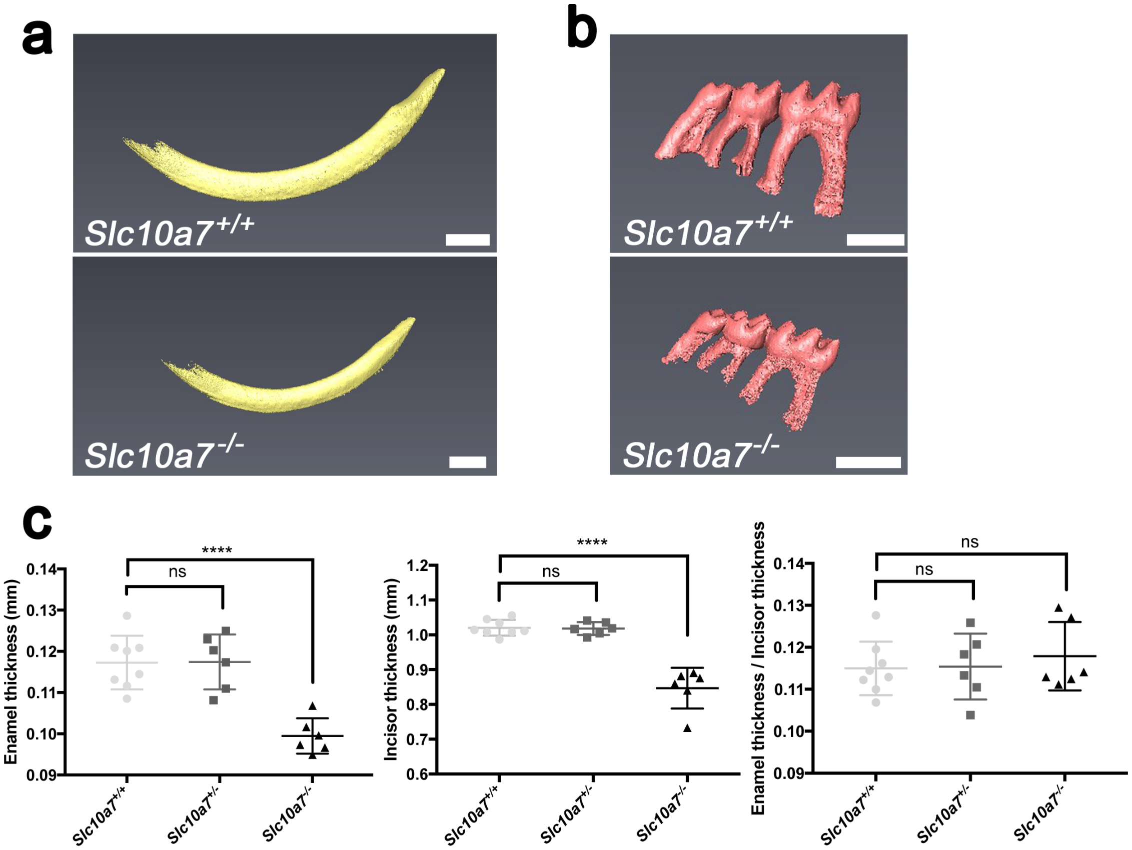

Supplementary Figure 6: **Tooth morphology was normal in *Slc10a7*$^{-/-}$ mice.**
a, b) Three dimensional reconstruction of lower incisors (**a**) and of lower molars (**b**) from µCT analysis of 8 week-old mouse skulls. Scales bars = 1 mm. **c)** Measurement of lower incisor enamel thickness and lower incisor thickness in 8 week-old mice. Data are expressed as mean±SD. n.s.: non-significant; ****: p≤0.0001 (two-tailed *t*-test). n = 7 (*Slc10a7*$^{+/+}$), n = 7 (*Slc10a7*$^{+/-}$) and n = 6 (*Slc10a7*$^{-/-}$).

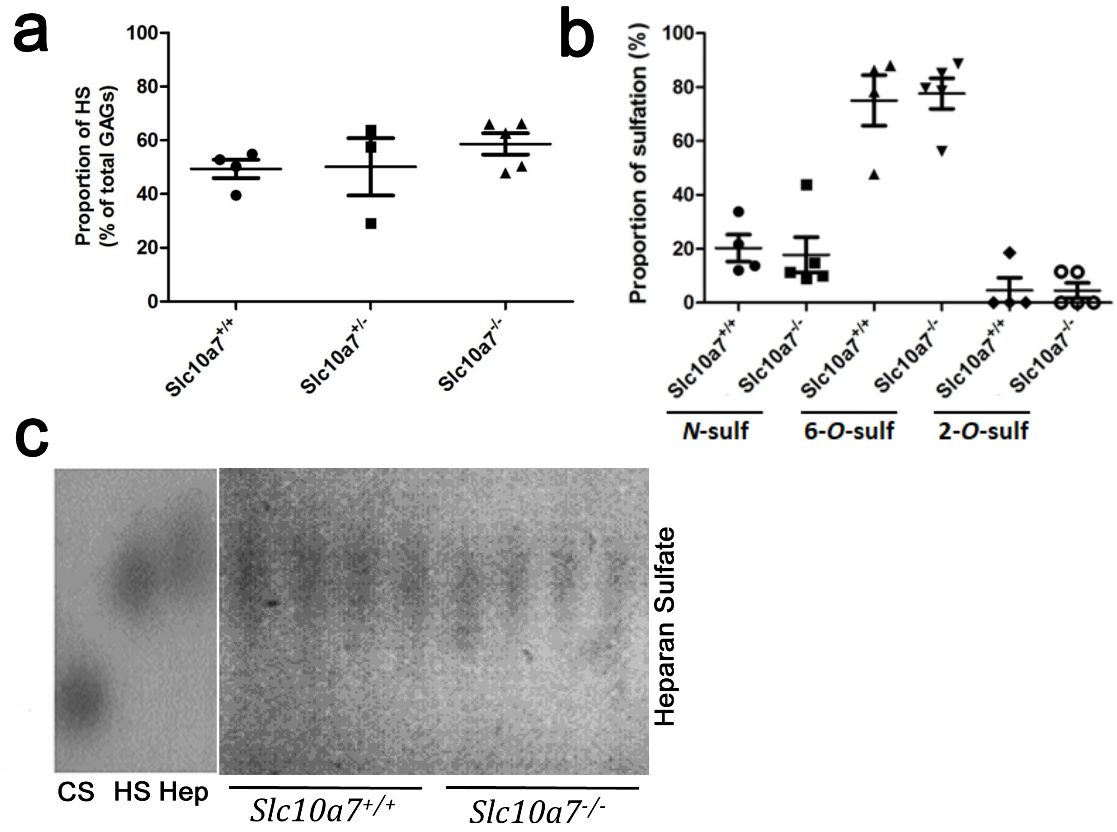

Supplementary Figure 7: GAG analysis in *Slc10a7*$^{-/-}$ mice.

a) *Slc10a7* deficiency has no impact in murine skeletal muscle heparan sulfate levels. Quantification of Total GAGs were extracted from thigh skeletal nuscle tissue of 10 day-old *Slc10a7*$^{+/+}$, *Slc10a7*$^{+/-}$*, and Slc10a7*$^{-/-}$ mice (n = 5) and quantified according to the DMMB procedure. Proportions of HS (% HS) were determined after CS digestion by Chase ABC followed by DMMB quantification. HS. Results are expressed as a percentage of HS in relation to the total sulfated GAGs. **b)** *Slc10a7* deficiency has no impact in specific sulfation of HS from mouse cartilage. Heparan sulfates (HS) from cartilage of 10 day-old *Slc10a7*$^{+/+}$, *Slc10a7*$^{+/-}$*, and Slc10a7*$^{-/-}$ mice (n = 5) were digested with a mixture of heparitinase I/II/III cocktail, and the resultant disaccharides were subjected to strong-anion exchange chromatography in a Propac column eluted by a NaCl gradient with post-column fluorescence labeling. Sulfated disaccharides ΔUA-GlcNS, ΔUA-GlcNAc6S, and ΔUA2S-GlcNAc were detected and identities were confirmed by elution times of corresponding commercial standards. Peak areas were measured and disaccharide proportions were expressed in % of the total sulfated disaccharide in each sample (n=5). **c)** *Slc10a7* deficiency has no impact in sizes of HS chains from mice cartilage. Heparan sulfates (HS) from cartilage of 10 day-old *Slc10a7*$^{+/+}$ *and Slc10a7*$^{-/-}$ mice (n = 5) were analysed by electrophoresis in 0.5 % agarose gel (1.5 mm) at 50V for 60 min then 100V -360 min (n = 4). 2.5 µg of HS were loaded to the gel. HS were visualized by DMMB staining. Standard CS, HS, and heparin chains were used as molecular weight size indicators. No significant size changes were detected between the two mice groups.

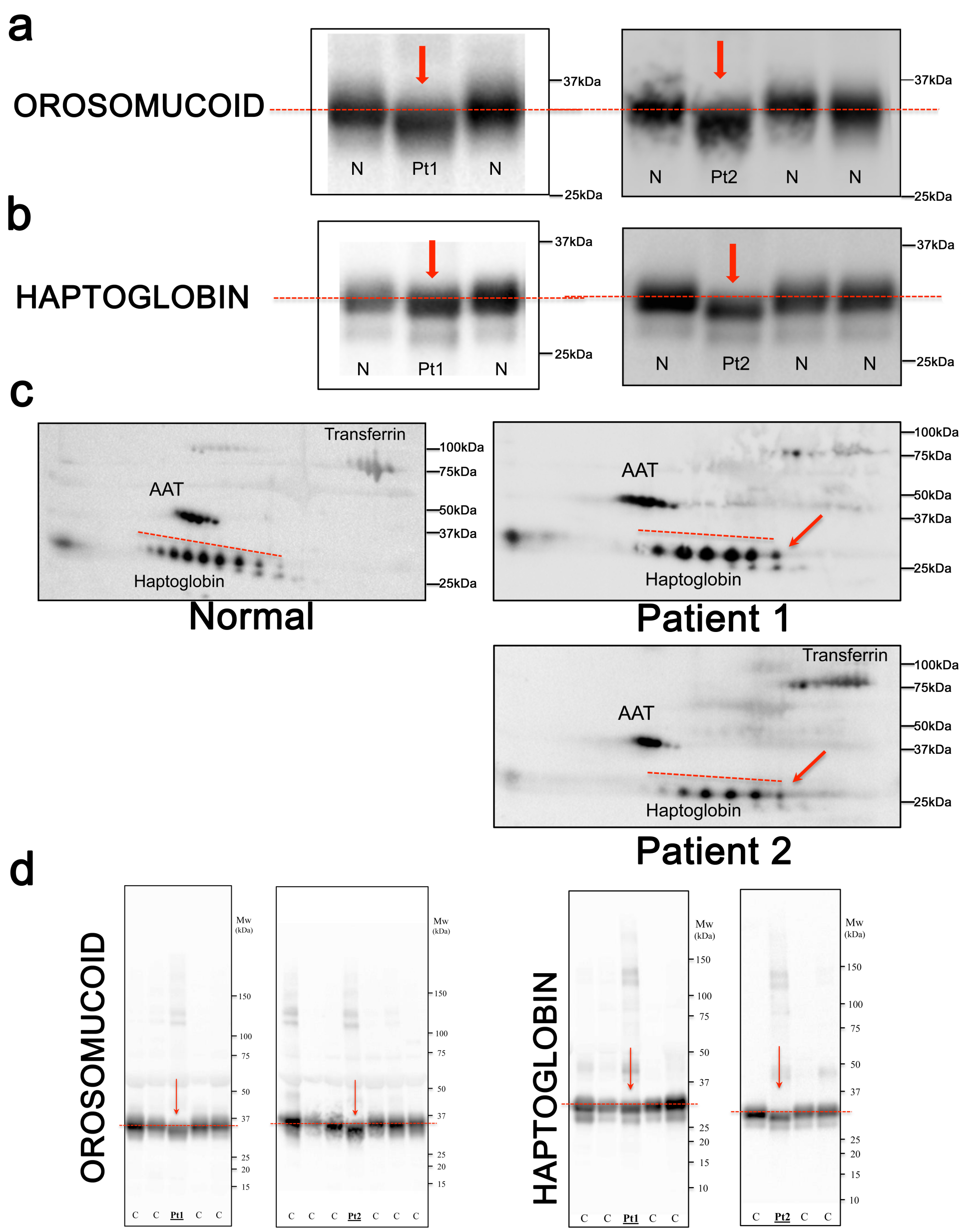

Supplementary Figure 8: *SLC10A7* deficiency causes modification of N-glycoprotein pattern.

A dried blood spot from 2 *SLC10A7*-deficient patients were analysed by electrophoresis for signs of a congenital disorder of glycosylation (CDG). Orosomucoid (**a**) and haptoglobulin (**b**) both showed a lower band in the *SLC10A7*-deficient patient (Pt1 and Pt2, red arrow) compared with control samples (N). The western blot images are cropped from gels which are provided for review in panel **d. c)** 2D electrophoresis of transferrin, Alpha-1-AntiTrypsin (AAT) and haptoglobin showing a shift of the far right glycoforms of haptoglobin (red arrow) in the *SLC10A7*-deficient patient samples (Patient 1 and Patient 2) compared with the control sample (Normal). **d)** Full unedited western blotting gels for panel **a**.

Primer names	Primer sequences
SLC10A7-1S	5'-GACTTTCCTGTCACTGGCTACTAC-3'
SLC10A7-1AS	5'-TTTCCAAGACTCACAAATTAGTTC-3'
SLC10A7-2S	5-'GGATTACAGGTGTGAACAAATAG-3'
SLC10A7-2AS	5'-AGCTATAAACAGCTTTACCAAAGG-3'
SLC10A7-3S	5'-TTTAGTAGGGATCTCCTTTCAGG-3'
SLC10A7-3AS	5'-CATAAGCCCTTTTGTATATAGTTGC-3'
SLC10A7-4S	5'-ATATGCCCTTATATTCATTTGCTC-3'
SLC10A7-4AS	5'-GAGTACACCTCCTTCTTTCACAAG-3'
SLC10A7-5S	5'-TTTGTAAGCAGTGACTAACTTTGC-3'
SLC10A7-5AS	5'-AACAAATCCAGCTTTCACTAAATC-3'
SLC10A7-6S	5'-AAGGGAATTTAGGTTTACAAAAGG-3'
SLC10A7-6AS	5'-CCTTTCTTAAAGGGTTGTAGATAGC-3'
SLC10A7-7S	5'-TGTTGAACTTAGAAAATTAGAGGTAAC-3'
SLC10A7-7AS	5'-TATCCTTTCTCTCAACTGCTCTG-3'
SLC10A7-8S	5'-ATGTCTTTCTCCTGTCAGAATCAC-3'
SLC10A7-8AS	5'-TACGATATAAGTGTAAAGCCGATG-3'
SLC10A7-9S	5'-AATGATTCTTTCAATTTAGCAAGG-3'
SLC10A7-9AS	5'-AAACGTGTATAGTGGCATAGTAGC-3'
SLC10A7-10S	5'-CTCCTTTTCATGGATAAATGACTC-3'
SLC10A7-10AS	5'-CATTCCATTTAATTTGAAGAGTTC-3'
SLC10A7-11S	5'-TTGATGGAGTATGCATTTAATTTG-3'
SLC10A7-11AS	5'-TTAAAATCTTTAACCTCAACCCTC-3'
SLC10A7-12S	5'-TTATTGGTTATATGATGCCATCTG-3'
SLC10A7-12AS	5'-AAATAAAATATGCATTGAGGCAAC-3'
SLC10A7-cDNA-7S	5'-TTTCTCAGCTTTTTATGACTGTTG-3'
SLC10A7-cDNA-11AS	5'-GGTAGATGAGCAAGGGTACAGATA-3'

Supplementary Table 1: List of primers used in this study.
